# Ocean Romance: Japanese Treefrogs Exploit Coastal Pools for Breeding Under Salinity and Habitat Constraints

**DOI:** 10.1002/ece3.73744

**Published:** 2026-05-28

**Authors:** Kyongman Heo, Deyatima Ghosh, Kevin R. Messenger, Siti N. Othman

**Affiliations:** ^1^ Herpetology and Applied Conservation Lab, College of Life Sciences Nanjing Forestry University Nanjing Jiangsu People's Republic of China; ^2^ Centre for Urban Ecology, Biodiversity, Evolution and Climate Change (CUBEC) JAIN (Deemed‐to‐be) University Bengaluru Karnataka India; ^3^ School of Natural Sciences and Engineering, National Institute of Advanced Studies Bengaluru Karnataka India; ^4^ Laboratory of Animal Behaviour and Conservation, College of Life Sciences Nanjing Forestry University Nanjing Jiangsu People's Republic of China

**Keywords:** breeding, coastal pools, *Dryophytes japonicus*, microhabitat, salinity, topography, water quality

## Abstract

Understanding amphibian microhabitat selection in coastal landscapes offers insight into patterns of local ecological adaptation in coastal island populations. This study examined whether coastal pools can sustain populations of the Japanese Treefrog, *Dryophytes japonicus* , a species whose coastal breeding ecology remains poorly understood. Additionally, we tested the prediction that salinity strongly influences breeding site use. We combined a meta‐analysis of 95 published studies with single‐time‐point field surveys of 810 tadpoles from 84 coastal pools across five topographically varied sites on Jeju Island, Republic of Korea. Using random forest and partial dependence analyses, we assessed eight environmental factors shaping tadpole occurrence. Across all studies reviewed, publication patterns showed no thematic or temporal bias across decades. However, more than 70% of studies focused on agricultural habitats, while fewer than 5% examined natural coastal pools, revealing a major knowledge gap. Empirical field data integrated with statistical modeling showed that, although salinity was the focal predictor of our study, distance to the coastline emerged as the strongest determinant of tadpole occurrence, with greater occurrence in pools positioned farther inland. The species consistently bred in shallow, low‐salinity pools located away from both the forest edge and the immediate shoreline, revealing clear spatial filtering within the coastal landscape. Dissolved oxygen also contributed to tadpole occurrence, particularly in coastal pools, underscoring the importance of well‐oxygenated water for successful breeding. Together, these results provide ecological insight into how *D. japonicus* persists in coastal landscapes under multiple environmental constraints.

## Graphical abstract

Topographical features, including elevation and the distance of pools from the coastline and forest, influenced breeding‐site use in coastal landscapes. Pool characteristics such as area and depth, together with abiotic conditions particularly salinity, as well as dissolved oxygen, and temperature, further shaped breeding‐site suitability and patterns of tadpole occurrence. These findings demonstrate how multiple environmental gradients structure the coastal breeding ecology of *Dryophytes japonicus*.

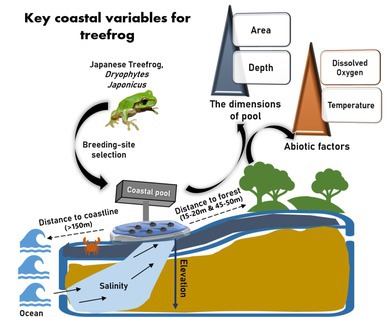

## 1 Introduction

Studying microhabitat selection at a local scale is essential to understand species‐specific requirements that enable amphibians to persist in changing environments. The high sensitivity of amphibians to environmental change, philopatric behavior, and limited dispersal also make them reliable bioindicators of habitat quality. Selection of breeding microhabitat is a key determinant of amphibian survivorship (Pettitt et al. 2018), because larvae are restricted to breeding pools (Gargaglioni and Milsom 2007), and are therefore unlikely to move autonomously from their natal sites (Rudolf and Rödel 2005). Hence, it is important for parents to use breeding sites that enhance larval survival (Wells 2010; Yu and Guo 2013). However, fluctuations in the fundamental parameters associated with microhabitat characteristics of amphibians, such as water quality (Budzik et al. 2014), could determine successful breeding and reproduction of amphibian populations, both in terrestrial and aquatic habitats landscapes (Beebee and Griffiths 2005). Especially, amphibians possess specific microhabitat requirements within a small‐scale habitat niche (Dubos et al. 2022), due to their high sensitivity to environmental changes, unique anatomy, highly philopatric behavior, and limited dispersal capacity, contributing to the extirpation of populations, both at local and global scales (Arroyo‐Rodríguez et al. 2017; Edwards et al. 2019; Seebacher and Alford 2002; Sodhi et al. 2008).

While the loss of terrestrial habitats is the primary concern for amphibians, aquatic breeding environments are also increasingly affected by anthropogenic factors, particularly in coastal systems. Coastal habitat degradation (Unglaub et al. 2018) and environmental change, including pollution (Schiesari et al. 2007), desiccation (Skelly 1996), and climate warming (Li et al. 2008) can influence breeding phenology. For instance, changes in humidity and temperature altered the temporal pattern of breeding in treefrogs in South America (Forti et al. 2022), In addition, changes in water quality have been shown to affect the occurrence of *Dryophytes suweonensis* and *Pelophylax plancyi* (Borzée, Heo, and Jang 2018; Borzée, Kyong, et al. 2018).

Several studies have reported the relationship between amphibian population, richness, and community and changes in water chemistry (Brodman et al. 2003; Glooschenko et al. 1992; Kolozsvary and Swihart 1999). While information regarding coastal populations of amphibians remains limited, generally, changes in climatic factors such as temperature, rainfall and salinity are predominantly linked to the fluctuations of coastal environments (Anderson et al. 2014), rendering these regions risk‐prone. Salinity for instance, affects all the stages of life history in amphibians in coastal habitats, ranging from developmental rates (Chinathamby et al. 2006), hatchling productivity (Lorrain‐Soligon et al. 2024), osmotic regulation (Karraker and Gibbs 2011), pathogen infection rates (Hall et al. 2020), and predation behavior toward the amphibians (Squires et al. 2008). However, the tolerance of amphibians toward salinity also varies across species (Hopkins and Brodie Jr 2015). At the time of writing, only 2% of global amphibian species are known to breed in saline environments (Hopkins and Brodie Jr 2015). Undoubtedly, the sensitivity of amphibians to saline environments provides an excellent model for investigating the potential of coastal habitats as breeding sites. This information will be integral for gaining insights to mitigate further degradation and preserve the focal species biodiversity.

*Dryophytes japonicus* is a hylid anuran distributed in Northeast Asia and is one of the most common amphibians in inland South Korea (Park et al. 2013) and has recently been reported to breed in coastal habitat from the island landscape of Jeju South Korea (Heo et al. 2019). The species inhabits a diversity of habitats, ranging from lentic to lotic water bodies and wetlands (Borzée et al. 2019; Borzée and Jang 2015; Koo 2019), However, in Jeju Island, where the composition of the habitats is mainly basalt, and due to the absence of rice paddies, the species breeds in small puddles found in dry streams and low‐lying pools along the coastline (Koo et al. 2018). The topography of coastal wetlands is usually composed of small pools, with low water volumes and fluctuating water quality such as pH, dissolved oxygen, salinity, total dissolved solids, and conductivity, creating a constant stressor for amphibian spawning and development (Lorrain‐Soligon et al. 2024). In addition, vernal pools located on coastal cliffs are directly exposed to the sun and predator risk. As a consequence, eggs and tadpoles encounter a high risk of desiccation from heat, as well as falling prey to predators such as fish (Laurila and Aho 1997), aquatic insects (Ohba 2009), and crabs, which are common in vernal pools near the coasts (Gray and Christy 2000).

Given that *D. japonicus* is a habitat generalist capable of thriving in diverse landscapes (Kim et al. 2019), we hypothesize that coastal pools can sustain *D. japonicus* populations. Here, we clarify the breeding behavior and ecology of *D. japonicus* by synthesizing published evidence and single‐time‐point field surveys to evaluate how tadpole occurrence varies with pool salinity, morphology, and surrounding terrestrial habitat. Although salinity may be relevant, we predict that pool morphology and adjacent terrestrial habitat primarily influence breeding site use, as inferred from tadpole occurrence. We tested this prediction by modeling tadpole occurrence in relation to pool and surrounding habitat features.

## 2 Materials and Methods

### 2.1 Literature Synthesis for *D. japonicus* Breeding Ecology

To gain a scientific understanding of *D. japonicus* breeding behavior and habitat requirements, we identified common patterns or trends in the breeding behavior and ecology of *D. japonicus* across different studies and geographic regions within the entire species range in Northeast Asia through a meta‐analysis. For Korean literatures, within the timeframe between 1931 and 2023, covering the oldest to the most recent relevant publications we found (Table S1). We reviewed relevant scientific literature and classified the studies into specific categories. Searches were conducted using international databases including Web of Sciences (https://www.webofscience.com/wos/), Google Scholar (https://scholar.google.com/), Scopus (https://www.scopus.com/), and Korean database platform DB pia (https://www.dbpia.co.kr/). To streamline the search with our targeted themes, we used Booleans logic (Boole 1854), using logical operators such as AND and OR to target species‐specific results. Because the Japanese treefrog was formerly known as *Hyla japonica* (Dufresnes et al. 2016), we conducted searches for both the updated name and its synonym. Each keyword was therefore searched in combination with both species' names separately (e.g., “ *Dryophytes japonicus* ” AND “breeding”, “ *Hyla japonica* ” AND “breeding”; “ *Dryophytes japonicus* ” AND “behavior”, “ *Hyla japonica* ” AND “behavior”). In total, 13 thematic keywords were used in this manner: “breeding”, “behavior”, “general ecology”, “predation”, “environment”, “spawning”, “habitat”, “calling”, “larvae”, “hibernation”, “water quality”, “salinity”, and “diet”. This procedure ensured comprehensive coverage of the species ecological literature under both nomenclatures. To classify the ecological literature, we assigned specific keywords to distinct ecological subdisciplines. Studies mentioning breeding or spawning were categorized under reproductive ecology, while those addressing predation or diet were placed in trophic ecology. Papers focused on environment or habitat were classified as habitat ecology, whereas those examining calling were grouped into behavioral ecology. References to hibernation were assigned to physiological ecology. Publications that did not clearly align with any of these subfields were placed into a separate category of general ecology. This approach ensured that each keyword was linked to a well‐defined ecological subdiscipline, while also providing a distinct category for broader studies.

We screened the first 30 search results, as results beyond this point showed a decline in relevance. After filtering the search results, we selected publications with keywords in the title if they were relevant to our study, and we read the abstract and added the publication if it matched the designated themes in our study. Our search initially identified 2880 publications. Although we considered 13 themes in the preliminary classification, some related categories such as water quality and salinity were merged for clarity, resulting in 10 final themes, including “behavior,” “breeding,” “ecology,” “hibernation,” “pathology,” “morphology,” “predation,” “conservation,” “water quality and salinity,” and “diet.” Each theme encompassed potential factors affecting breeding; for instance, “pathology” included diseases that reduce fertility, and thereby influenced breeding success.

Finally, we analyzed publication trends for *D. japonicus* from 1931 to 2023 by quantifying the number of studies within each ecological theme and habitat type to evaluate the distribution of research effort. We summarized temporal patterns using descriptive statistics and visualized changes in research focus across decades through bar charts and line plots. To assess whether specific themes such as breeding, ecology, behavior, morphology, hibernation, pathology, predation, conservation, and water quality/salinity were disproportionately represented across habitats or historical periods, we conducted Chi‐square tests of independence based on contingency tables of themes, habitat types, and decades. All metadata analyses were performed in R v.4.3.3 (R Core Team 2024) using the packages dplyr, ggplot2, and stats.

### 2.2 Empirical Field Data: Study System

**FIGURE 1:**
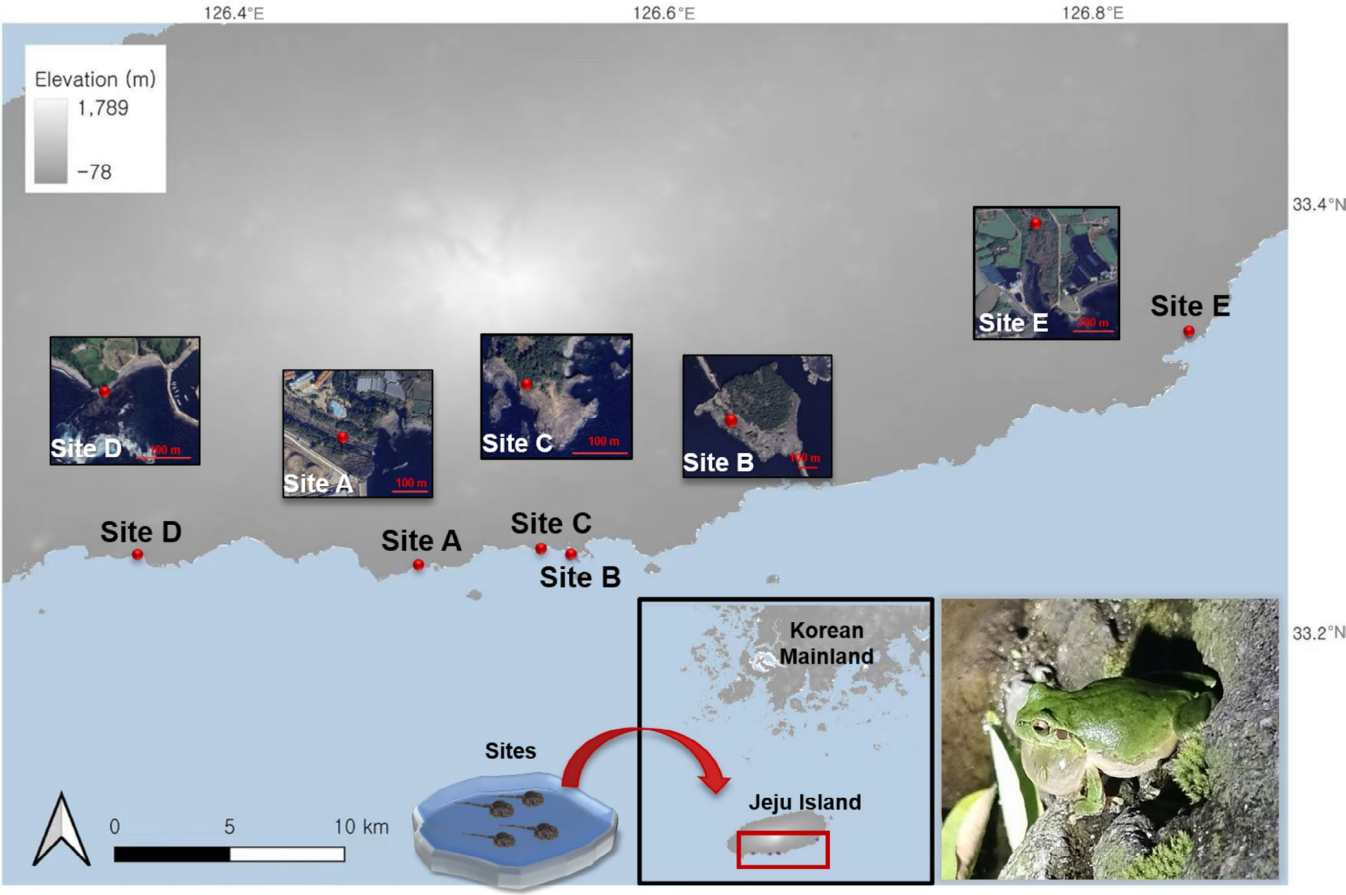
Coastal breeding sites of *Dryophytes japonicus* surveyed along the southern shoreline of Jeju Island, Republic of Korea. The five sites (A–E) span a range of coastal topographies, each containing natural pools where tadpoles' occurrence was recorded. Insets show aerial imagery of each site location for geographic context. The inset panel includes a photograph of an adult *D. japonicus* from Jeju Island, captured on Site E by Heo et al. (2019). Physical characteristics of all surveyed pools, such as pool area, depth, distance to coastline, and surrounding vegetation are provided in Table S2. Map is generated in QGIS v3.12.3 (QGIS Development Team 2020).

The surveyed area was located within a wetland at Seogwipo, the southern part of Jeju Island (33° E, 126° N; see map in Figure 1). Jeju Island is volcanic with a distinctive subtropical marine ecosystem, and a mean annual temperature of 16.7°C, setting it apart from the Korean mainland (Kang et al. 2013). The climate of the island was majorly influenced by monsoon winds, with the highest precipitations concentrated in the summer, between 597.3 and 792.7 mm, and a mean temperature of 24.5°C (Jeju Regional Meteorological Administration 2022). Specifically, the southern region of the island was directly affected by climate fluctuation and sea level change, resulting in the formation of basaltic wave cliffs and creating subtropical‐like environments (Kim 2007).

The coastal pools serving as the breeding sites for *D. japonicus* larvae included ponds of various sizes, positioned on the basaltic wave cliffs spanning across five sites (Figure 1): (1) Gangjeongcheon stream (Site A), (2) Saeseom Island (Site B), (3) Storm Hill (Site C), (4) Baksugijeong cliff (Site D), and (5) Cheonmicheon stream (Site E). Site A is characterized as a riverine area, composed of streams that flowed through the city center, Seogwipo City. Site A rises only 19 m above sea level, allowing waves to bring in salt. Unlike most cement‐managed island streams, it holds clear spring water year‐round and forms a well‐developed estuary (Kim et al. 2024). Site B is an isolated and small island located in the south of Jeju Island, which was fed by groundwater springs and is generally marshy along the edge of the island (Figure S1). The present coastal pools tadpoles were primarily distributed on the southeastern side of site B, composed of low‐elevated vertical cliffs (10 m above sea level). Site C is characterized as a basaltic, fractured cliff area with saline pools lining at the edges. The elevation of this site ranging between seven and 20 m, and the site is divided into non‐vegetated and vegetated sections. The group of pools was found at the end of a wave‐cut platform, within the southwestern Jeju Island. Site D consists of a 100 m vertical cliff, with stagnant water pools forming in basalt burrows just below the cliff. The minimum elevation, at the base of the Baksugijeong cliff, ranged from 3 to 5 m. In addition, the site is one of the closest proximities to the coastline compared to the other site. Unlike the other sites, Site E in southeastern Jeju Island was a brackish stream, where discharge from a nearby fish farm mixed with seawater, resulting in elevated salinity levels (Figure 1).

### 2.3 Species Model

*Dryophytes japonicus* is the most common and widespread of the three *Dryophytes* species in the Korean Peninsula, breeding from April to July and occurring throughout the region, where it primarily breeds in rice paddies, except for some island (Borzée, Kyong, et al. 2018). However, on Jeju Island, recent findings indicated that the species inhabited high‐salinity brackish pools, and bred in small pools at stream edges or in low‐lying coastal pools (Heo et al. 2019). The species is potentially having a diverse range of breeding sites, contributing to their widespread distribution in various ecosystems, especially in Korean Peninsula (Kim et al. 2019), and therefore represents the best model system for studying the effect of dynamic changes within coastal ecosystems on the breeding microhabitat selection of the species.

### 2.4 Coastal Variables

We measured tadpole occurrence and nine ecological variables relevant to the coastal environment and the breeding requirements of *D. japonicus* (Table S2). These variables included: (1) Salinity and habitat type; we measured salinity in each pool using a handheld meter and classified the pools into brackish and non‐brackish type, depending on the salinity levels based on the threshold range for brackish criteria proposed by Hopkins and Brodie Jr (2015): concentration < 1 ppt represented non‐brackish water, and concentration > 1 ppt represented brackish water. (2) Pool area (m^2^); for circular pools, we measured the diameter (the distance across the widest point) using a measuring tape. The radius was obtained by dividing the diameter by two, and the surface area of each pool was calculated using the circular area formula;

$$\text{Surface area}=\pi \;x\;{\text{radius}}^{2}$$

(3) Temperature (°C), (4) dissolved oxygen (DO) (mg/L), with both variables measured 10 cm from the edge of each pool using the DO meter (YK‐2001PHA, LUTRON ELECTRONIC ENTERPRISE CO. LTD.), (5) distance of the pool from the coastline (m); we marked the GPS point of each pool, and then we measured the distance of the pool from the shoreline using the measuring tool on Google Earth's tool (Google 2025), (6) distance of the pool from the forest (m); we marked the GPS point of each pool and we measured the distance of the pool from the closest natural vegetation using the measuring tool on Google Earth's tool, (7) elevation (m); we marked the GPS point of each pool and measured the elevation in meters above sea level, using Google Earth's tool. (8) depth of the water (m); we measured the deepest part of the pool (usually at the center), using a ruler and a tape measure.

### 2.5 Empirical Data Collection

Data collection was conducted over 2 years (2021–2022), during the breeding season of *D. japonicus* on the Korean Peninsula (May–July in each year). We visited pools repeatedly during the study period to confirm tadpole presence and site conditions. However, the analyses presented here were based on a single survey occasion per pool, for which tadpole occurrence and environmental variables were recorded simultaneously. These data should therefore be interpreted as site‐specific measurements collected during the survey period rather than as continuous records across the breeding season. First, we defined each site by maintaining sufficient distance between them, considering the concept of home range. To do so, we established five sites at least 200 m apart that contained pools where frogs could spawn, and randomly selected pools within the site area to determine the presence or absence of frogs. Amphibian home ranges are typically between 159 and 290 m (Semlitsch and Bodie 2003), for instance, the home range of California treefrog, *Pseudacris cadaverina* , does not exceed 300 m (Dole 1974). For our study, we were essentially focused on the tadpole stage; hence there was minimum chance of pseudoreplication. Although a transect distance of > 200 m is theoretically ideal for tracking the home range of *D. japonicus* , we also accounted for the water flow conditions of each pool. Given their limited mobility, tadpoles were not expected to disperse beyond the pool boundaries. Each pool observed was at least 3 m away from each other without any steady flow of water between them. We recorded the presence or absence of treefrog tadpoles in each pool, which we used as an indicator of breeding site use rather than direct evidence of adult oviposition preference. In total, we surveyed 12 pools in site A, 51 pools in site B, nine pools in site C, seven pools in site D, and four pools in Site E.

### 2.6 Statistical Analyses for Empirical Data Collection

All statistical analyses for the empirical field dataset were conducted in R v.4.3.3 (R Core Team 2024). First, we explored relationships among topographical and environmental variables to assess correlations and identify important predictors for the study. To do so, we built a best‐fit decision tree using random forest analyses, based on the ensemble of decision trees generated by the algorithm. The random forest approach captures complex relationships between input features and the response variable thus provided a measure of feature importance, indicating which topographic features are most influential in predicting the response and understanding the data's underlying structure. Here, we conducted random forest analyses with 500 decision trees, and each tree being trained on a bootstrap sample of the training data. At each node of the decision tree, a random subset of three features was considered for splitting, contributing to the diversity of the individual trees. Additionally, out‐of‐bag (OOB) error estimation was used to provide an unbiased estimate of model performance. We used Mean Decrease Gini scores which indicate the decrease in Gini impurity when a feature is used to split the data within the random forest ensemble. These analyses were conducted using the package “randomforest” (Breiman 2001).

Following identification of the most important predictors, we generated partial dependence plots to visualize the marginal effect of each variable on tadpole occurrence while holding the others constant. These plots were used to interpret the direction and shape of predictor–response relationships, rather than to select the model family. We used the package pdp (Greenwell 2017) for these visualizations.

We performed a Principal Component Analysis (PCA) to reduce the dimensionality of the dataset and explore the importance of the variables using library vegan (Oksanen 2010), factoextra (Kassambara 2016), corrr (Kuhn et al. 2020), and ggcorrplot (Alboukadel 2019). To explore the relationship between the variables, we generated a correlation matrix to inform predictive modeling, using the Hmisc (Harrell and Dupont 2020) and corrplot (Wei and Simko 2017) packages. We also performed Kruskal Wallis analysis, to understand the difference between the environmental variables across the five populations. Except for temperature, none of the variables showed normality when tested using Shapiro–Wilk and Levene's test (Table S3), therefore, we used the non‐parametric form of ANOVA. We used packages FSA (Ogle et al. 2020), ggplot 2 (Wickham 2016). Furthermore, to understand how different environmental and topographic features might be impacting *D. japonicus* at the community level, we compared the total number of tadpoles between the pools across five sites using Kruskal Wallis. Normality assumptions were checked using Shapiro–Wilk (*W* = 0.803, *p* < 0.001) and Levene's test (*F* = 1.433, df = 4, *p* = 0.231).

To examine the effects of environmental and topographic variables on tadpole occurrence, we fitted a generalized linear model (GLM) with a binomial error distribution and logit link. Prior to analysis, we scaled all continuous predictor variables, and assessed multicollinearity using variance inflation factors (VIF < 2; Ghosh and Basu 2023). Because normality is not assumed for binomial models, we did not evaluate normality using Shapiro–Wilk tests. Instead, model fit was assessed by checking for overdispersion. We then used backward model selection based on the Akaike's Information Criterion (AIC), implemented using R function ‘step’, to identify the most parsimonious occurrence model. Model fit was further evaluated using Nagelkerke's pseudo‐*R* ^2^ and comparison with the null model. We used packages lmtest (Zeileis and Hothorn 2002), lme4 (Douglas Bates et al. 2015), rcompanion (Mangiafico 2020), and car (Fox and Weisberg 2018) for all analyses.

## 3 Results

### 3.1 Publication Trends

**FIGURE 2:**
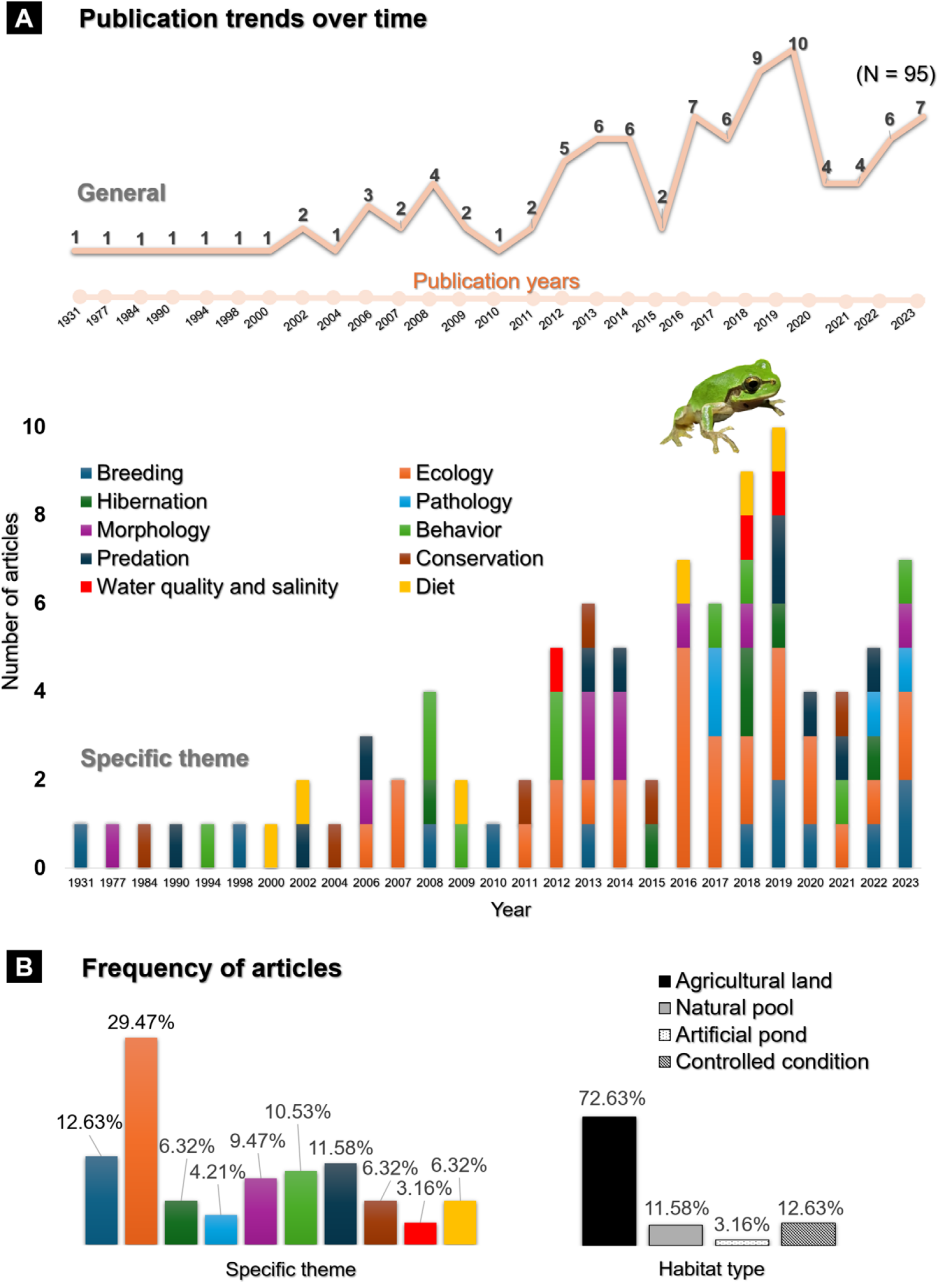
Publication trends on *Dryophytes japonicus* over the past 93 years. (A) Temporal distribution of 95 studies published between 1931 and 2023 across 10 ecological themes, showing an overall increase in research effort, particularly after 2010. (B) Frequency of studies conducted across four habitat categories, illustrating a strong bias toward agricultural systems (72.63%) and a pronounced scarcity of research in natural coastal habitats (< 5%).

Our review of 95 papers published between 1931 and 2023 revealed that, although several aspects of the breeding biology of *D. japonicus* have been studied, there remains a clear gap in research on their breeding behavior (Figure 2). Prior to the 21st century, the breeding behavior of this species was not well studied, with only six related studies published. However, research activity increased significantly after 2000. The publication trends showed a steady increase in interest in this species, with notable peaks in 2008, 2013, and 2019. After ecology, the most researched topics were breeding, predation, behavior, and morphology, with each topic having more than nine papers published over the past nine decades. It is important to note that the “behavior” theme covered broad activity patterns (i.e., calling, movement and foraging), whereas breeding and predation research targeted specific ecological processes such as reproduction and feeding interactions (Table S1). However, despite the surge in ecological studies since the 2010s, research explicitly examining water quality and salinity in *D. japonicus* remains scarce (Figure 2A). The categorization of breeding habitat in the 95 publications revealed that over 70% of the studies focused on agricultural lands, with 68 publications based in rice paddies and only one reporting the species from soybean fields. Only 11% of studies reported the species from forests and natural pools. Notably, we did not find any study on *D. japonicus* that explored the potential of coastal habitats, supporting the breeding of the species in coastal habitat.

Across the literature dataset, we found no significant association between ecological theme and habitat type (*χ* ^2^ = 22.49, *p* = 0.550; Table S4), nor between theme and period of publication in decade (*χ* ^2^ = 21.71, *p* = 0.560; Table S5). Habitat type also showed no significant temporal pattern across decades (*χ* ^2^ = 6.58, *p* = 0.516; Table S6). However, a one‐way Chi‐square test revealed strongly uneven habitat‐type frequencies (*χ* ^2^ = 117, df = 3, *p* < 2.2 × 10^−16^), confirming that studies on *D. japonicus* have been biased toward agricultural systems (Figure 2).

### 3.2 Correlation Structure Among Environmental Variables

Topographic variables showed diverse correlation patterns among themselves, with significant negative correlations between: (a) distance of the pool from the coastline and distance of the pool from the forest (*r* = −0.37; *p* < 0.001; Figure S2), and (b) distance of the pool from the forest and elevation (*r* = −0.34; *p* = 0.002; Figure S2). Meanwhile we identified significant positive correlation between: (a) salinity and distance of the pool from forest (*r* = 0.37; *p* < 0.001; Figure S2), and (b) area and depth of pool (*r* = 0.27; *p* = 0.02; Figure S2).

### 3.3 Variations in Habitat Properties Across Five Sites

**FIGURE 3:**
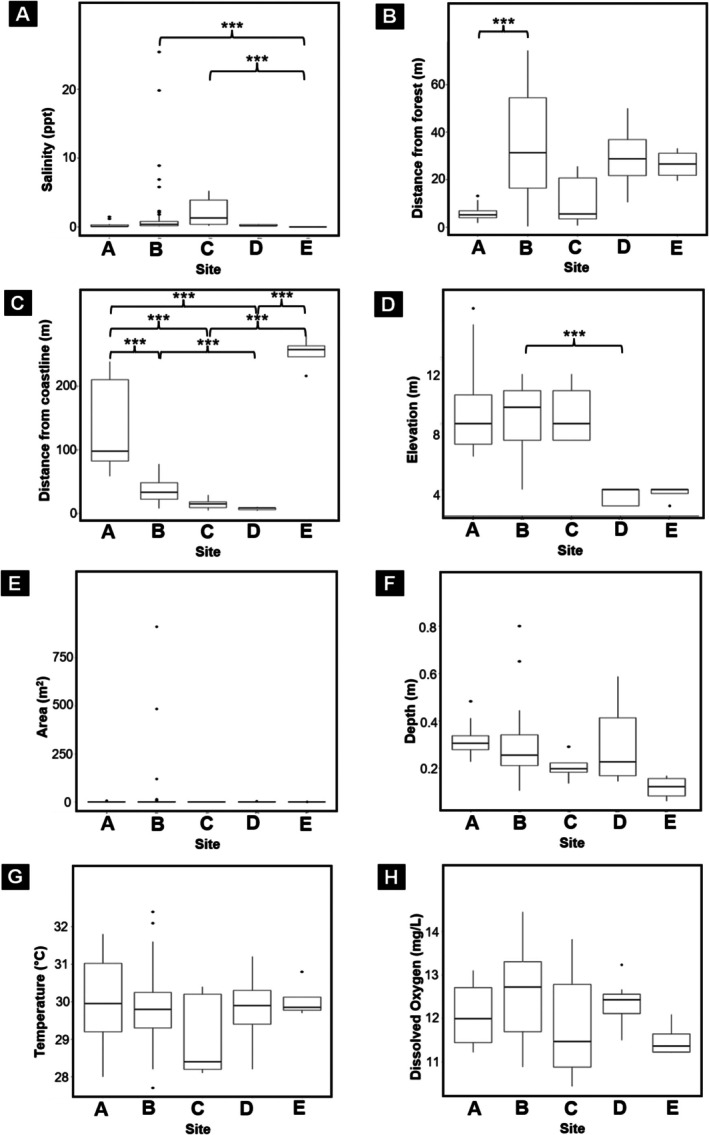
Kruskal–Wallis boxplots illustrating variation in coastal environmental variables across the five breeding‐pool sites on Jeju Island. Each panel (A–H) shows the distribution of one variable across Sites A–E: (A) salinity, (B) distance from forest, (C) distance from coastline, (D) elevation, (E) pool area, (F) pool depth, (G) temperature, and (H) dissolved oxygen. Significant pairwise differences among sites are indicated by asterisks, where *p* < 0.01 (***). These comparisons highlight spatial heterogeneity in abiotic and structural characteristics across coastal breeding pools.

We found significant among‐site variation in six coastal variables across the five study sites (Sites A–E), including salinity, distance from forest, distance from coastline, elevation, pool area, and pool depth (Figure 3). Kruskal–Wallis tests indicated significant site‐level differences in salinity (*χ* ^2^ = 22.348, df = 4, *p* < 0.001), distance from forest (*χ* ^2^ = 24.928, df = 4, *p* < 0.001), distance from coastline (*χ* ^2^ = 53.369, df = 4, *p* < 0.001), elevation (*χ* ^2^ = 28.122, df = 4, *p* < 0.001), pool area (*χ* ^2^ = 15.078, df = 4, *p* = 0.005), and pool depth (*χ* ^2^ = 18.248, df = 4, *p* = 0.001). In contrast, temperature (*χ* ^2^ = 5.006, df = 4, *p* = 0.286) and dissolved oxygen (*χ* ^2^ = 8.052, df = 4, *p* = 0.089) did not differ significantly among sites. Pairwise differences are shown in Figure 3.

### 3.4 Key Coastal Variables Influencing Tadpole Occurrence

Model selection identified salinity as an important predictor of tadpole occurrence, although it was not the sole determinant. The most parsimonious occurrence model included salinity, temperature, pool area, depth, distance from forest, and sampling site (AIC = 89.520; Table S7).

Tadpole occurrence did not differ significantly among sites (Kruskal–Wallis *χ* ^2^ = 3.875, df = 4, *p* = 0.423), despite site‐level variation in salinity, distance from forest, pool depth, and elevation (Figure 3). In the final occurrence model, salinity (*β* = −0.482, *p* = 0.014) and pool depth (*β* = −0.418, *p* = 0.032) were significant negative predictors of tadpole occurrence, indicating that higher salinity and deeper pools reduced occurrence probability. Distance from forest showed a marginal effect (*β* = 0.410, *p* = 0.104), whereas dissolved oxygen (*β* = 0.247, *p* = 0.162), temperature (*β* = 0.025, *p* = 0.879), pool area (*β* = −6.149, *p* = 0.223), and elevation (*β* = −0.115, *p* = 0.639) were not significant predictors. Distance from the coastline was excluded from the final model due to collinearity (GVIF = 2.763). The occurrence model explained a modest proportion of variation in tadpole occurrence (Nagelkerke's *R* ^2^ = 0.207; *χ* ^2^ = 19.205, *p* = 0.058).

### 3.5 Model‐Predicted Occurrence Patterns Across Environmental Gradients

**FIGURE 4:**
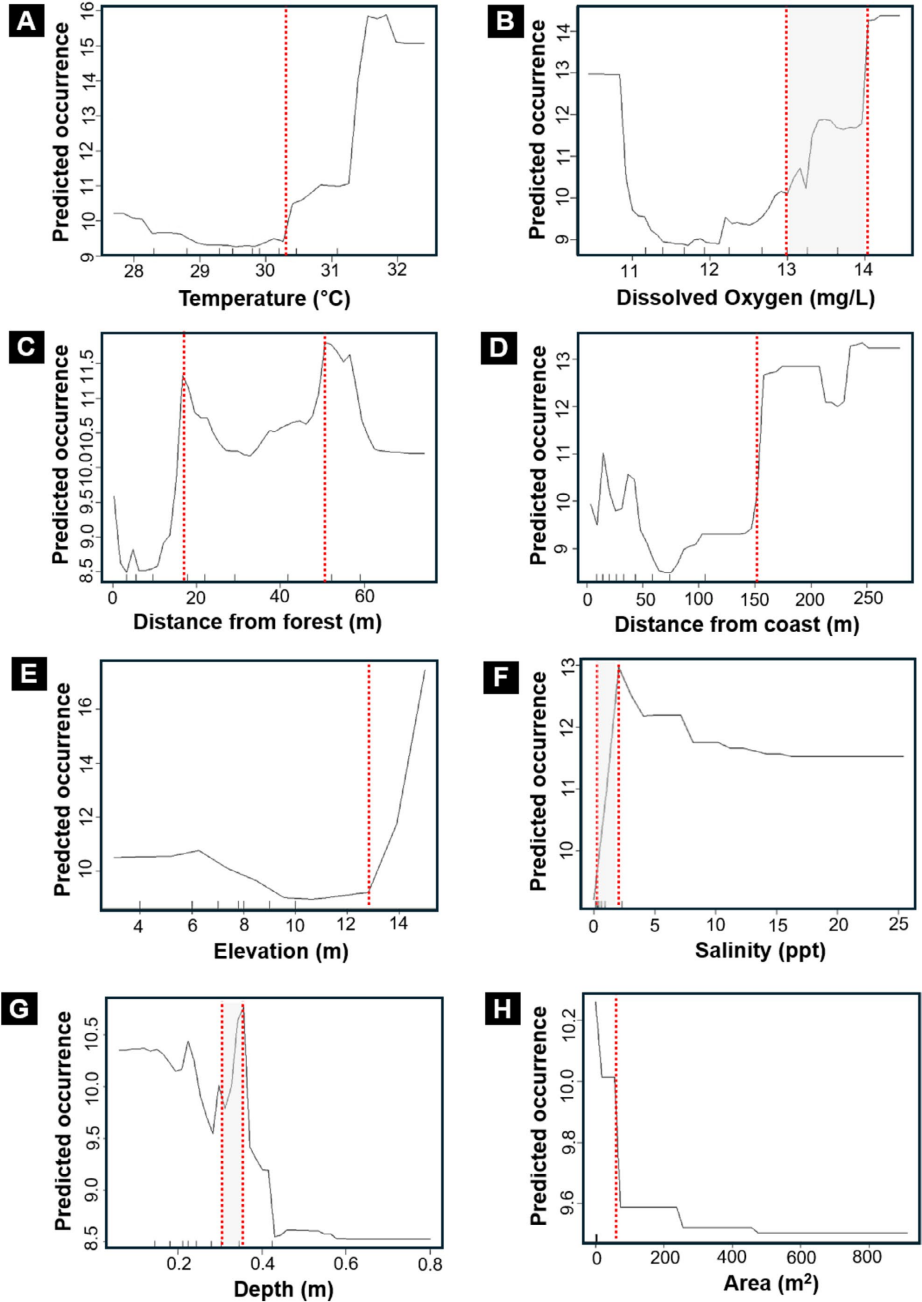
Predicted occurrence probability of tadpoles in coastal breeding pools based on the random forest model. The *x*‐axis shows the observed range of each variable, and the *y*‐axis shows the fitted probability of tadpole occurrence. Tick marks represent the distribution of observed data values. Red dashed lines indicate key inflection points where predicted occurrence changes. Higher predicted occurrence was associated with temperatures above 30°C (A), dissolved oxygen levels above 13 mg L^−1^, although the dissolved oxygen response was non‐linear and bimodal (B). The relationship with distance from forest also showed a bimodal pattern, with higher predicted occurrence at pools located approximately 15–20 and 45–50 m from the forest edge (C). Pools located more than 150 m from the coastline were associated with higher predicted occurrences (D). The occurrence of tadpoles also increased at elevations above 12 m (E), and at pool depths around 0.25–0.30 m (G). Whereas reduced predicted occurrence was associated with salinity exceeding 2.5 ppt (F) and larger pool areas (H).

Our predictive modeling highlighted several environmental variables that could be important for explaining tadpole occurrence in coastal breeding pools. Partial dependence plots showed that tadpole occurrence tended to increase at higher temperatures above 30°C (Figure 4A), and at dissolved oxygen levels above 13 mg/L, although the response to dissolved oxygen was non‐linear and bimodal (Figure 4B). In terms of topography, the relationship with distance from forest also showed a bimodal pattern, with higher predicted occurrence at pools located between 15–20 m and 45–50 m from the forest (Figure 4C). While pools located more than 150 m from the coastline were associated with high predicted tadpole occurrence (Figure 4D). A noticeable increase in occurrence was observed above 12 m elevation (Figure 4E). Salinity above 2.5 ppt however, was associated with reduced tadpole occurrence (Figure 4F). In terms of the dimensions and shape of the pools, the highest predicted occurrence was observed at depths of 0.25–0.3 m (Figure 4G), whereas larger surface areas (> 1530 m^2^) were associated with lower occurrence (Figure 4H).

**FIGURE 5:**
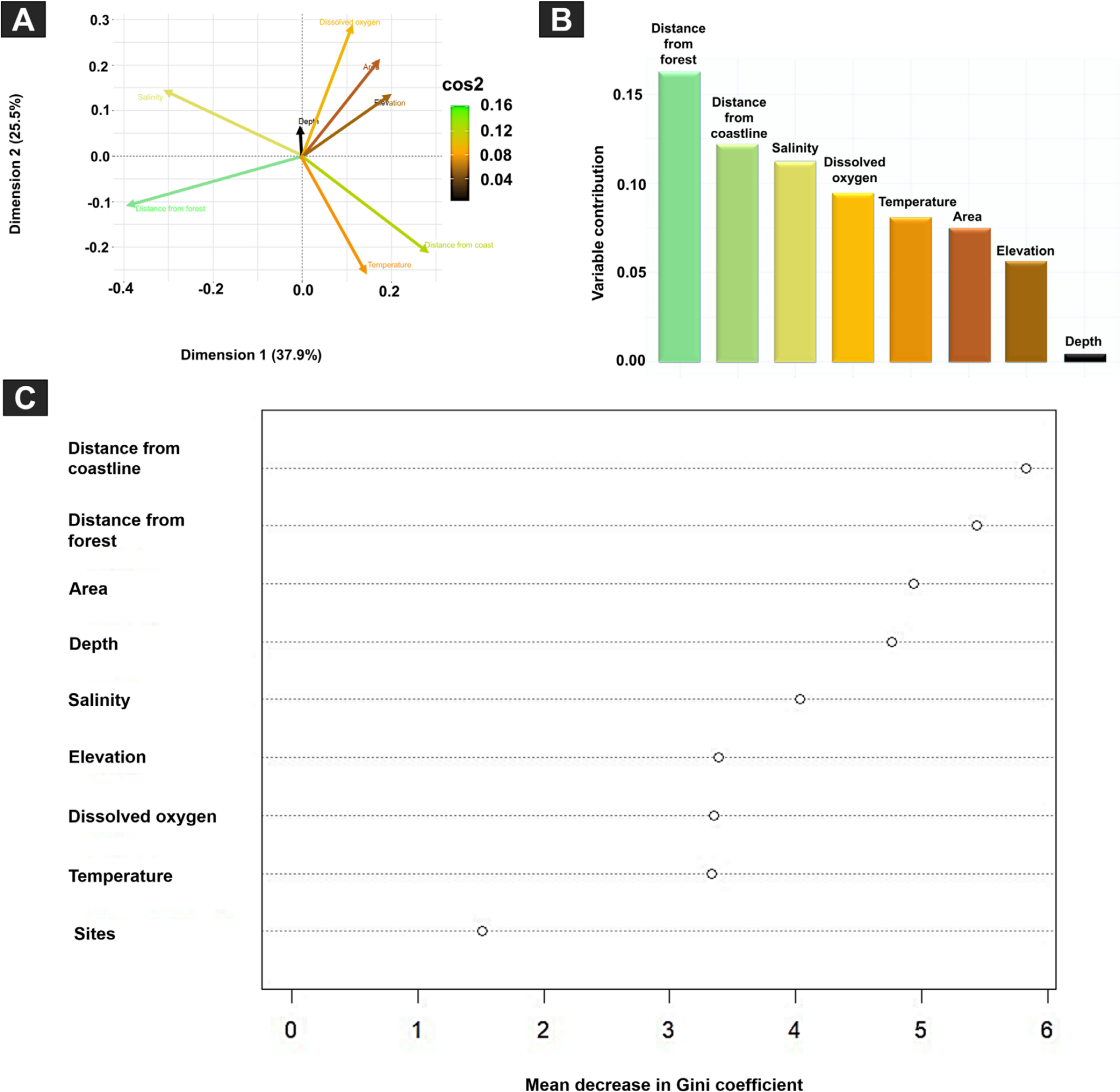
Multivariate and machine‐learning assessment of environmental variables influencing the occurrence of *Dryophytes japonicus* in coastal breeding pools. (A) Principal Component Analysis (PCA) biplot showing the major coastal variables contributing to variation across pools; vector length indicates strength of association, and cos^2^ coloring reflects variable quality of representation on PCA axes. (B) Variable importance scores from the random forest model, expressed as percentage contribution to predictive accuracy. (C) Mean Decrease Gini values indicating the relative importance of each variable in classifying tadpole occurrence, with higher values corresponding to stronger influence on model performance.

In addition, PCA indicated that the distance of the breeding pool from the forest and the coastline were the most important variables, followed by salinity, dissolved oxygen, temperature, pool area, elevation, and depth (Tables S8 and S9; Figure 5; see panels A and B). Furthermore, the predictions of the random forest model on unseen data points (the out‐of‐bag samples) had an error rate of 25%, indicating that approximately 75% of the out‐of‐bag predictions were correct (Table S10; Figure 5C). The distance of the breeding pool from the coastline emerged as the strongest predictor of tadpole occurrence (MeanDecreaseGini = 5.835; Table S11; Figure 5) and may also interact with other variables in shaping distribution patterns. The distance of pools from the forest accounted for the next most important predictor (MeanDecreaseGini = 5.438), followed by pool area (MeanDecreaseGini = 4.941), pool depth (MeanDecreaseGini = 4.762), salinity (MeanDecreaseGini = 4.040), elevation (MeanDecreaseGini = 3.393), dissolved oxygen (MeanDecreaseGini = 3.348), and temperature (MeanDecreaseGini = 3.335). Survey site identity had the lowest predictive importance (1.508; Figure 5). Together, our results emphasize the dominant role of topographic context (coastal and forest distance), pool structure, and salinity in shaping tadpole occurrence.

## 4 Discussion

This study provides evidence for flexibility in breeding site use by *D. japonicus* and suggests that coastal pools in island landscapes could serve as potential habitat for sustaining this species. However, the extent of this use remains unknown. In our study sites, predicted tadpole occurrence was above 60% at 0% salinity, but dropped to less than 25% at salinity levels of 0.75% and above (Figure 4). This is broadly consistent with a previous study, reporting *D. japonicus* tadpoles in pools with salinities of around 1% (Heo et al. 2019), but lower than salinity values reported for more salt tolerant coastal species such as *Fejervarya cancrivora* and *Pelophylax ridibundus* (Hsu et al. 2012; Mollov 2020). Although salinity is one of the most important factors limiting the local occurrence and density of tadpoles in coastal areas, other abiotic and topographic factors also have potential to influence breeding biology, especially breeding site use inferred from tadpole occurrence, with probable implications at the community level in *D. japonicus* (Figure 5).

To date, 140 amphibian species have been observed in saline habitats (ranging from tidal mangrove swamps to inland freshwater habitats contaminated with road deicing salts) and this represents only 2% of all known species (Hopkins and Brodie Jr 2015), indicating the general notion that anurans are salt‐sensitive and prefer freshwater (Figure 2). Coastal landscapes are fragile and unique at the same time; the options for ideal spawning sites are limited both by water quality, mostly salinity, as well as the lack of other suitable habitats such as rice paddies and natural pools. The vernal pools receive water only during periods of rainfall, and heavy rain can force tadpoles out of the pools and into the sea. There is not enough evidence to suggest that *D. japonicus* is a salt‐tolerant species; however, the occurrence of tadpoles in these coastal pools suggests that the species can use coastal habitats for breeding, although the extent of this use remains to be explored. Compared with the exceptionally salt‐tolerant amphibian species, *Fejervarya cancrivora, Pelophylax ridibundus, and Buergeria japonica* (Hopkins and Brodie Jr 2015), *D. japonicus* does not appear to be strongly euryhaline. However, it may also be premature to interpret this species as unusually salt‐sensitive, as tadpoles were still recorded in several pools with moderate brackish conditions, suggesting some tolerance to elevated salinity.

*Dryophytes japonicus* prefers agricultural lands especially paddy fields for breeding (Figure 2). Adults breed in lotic or slow‐flowing water bodies, and then the species migrate to forested hills during the non‐breeding season between late August and September in mainland South Korea (Borzee et al. 2019; Sugimoto and Jiang 2008). Because Jeju Island's porous basaltic substrate cannot support rice paddies, the main breeding habitat available to *D. japonicus* consists of small coastal or vernal pools located near natural vegetation (Jeong 2014). As a result, the most viable spawning habitats on Jeju are the small vernal or coastal pools that form near the shoreline. Our findings support this ecological constraint, as tadpole occurrence was associated with pools situated close to the coastline and adjacent to natural vegetation, highlighting the importance of both aquatic and surrounding terrestrial microhabitats (Figure 4). The dependence of the species on the critical terrestrial habitat around the breeding pools is consistent with the ‘life zone’ (Semlitsch 1998), in which the integration of breeding pools and nearby upland habitat is essential for completing the amphibian life cycle. Additionally, this species is also known to occur in pools near forest edges (Roh et al. 2014), and even in mountain habitats (Naito et al. 2013), suggesting a degree of flexibility in tolerating a range of climatic and abiotic conditions. Our results further support this interpretation, showing that *D. japonicus* can occur and reproduce in some coastal pools with elevated salinity, although this should not be interpreted as evidence of broad salinity tolerance.

Increased salinity disrupts the ionic and osmotic homeostasis of larvae which are hyperosmotic relative to their environment (Ultsch et al. 1999; Brooks and Mills 2003). In addition to this, saline water holds 20% less dissolved oxygen than freshwater (Truesdale et al. 1955). Therefore, tadpoles inhabiting coastal pools need to be locally adapted to the coastal environment to survive the dual challenge of high salinity and low dissolved oxygen levels (Bell 2017). Increased allostatic load to maintain osmotic balance in high salinity environments would impair energy allocation to physical performance such as foraging and swimming and eventually delaying metamorphosis (Gomez‐Mestre and Tejedo 2003). Studies of various anuran species have shown that exposure to elevated salinity impairs tadpole growth ultimately reducing animal body size (Wu and Kam 2009). Smaller body size reduces post‐metamorphic survival (Gomez‐Mestre and Tejedo 2003), delayed sexual maturity (Semlitsch et al. 1988), and weakened cellular immune function in adults (Gervasi and Foufopoulos 2008). There is emerging evidence that such effects on individuals reduce recruitment and negatively impact the viability of breeding populations (Berven 1990). Further, dissolved oxygen in breeding pools is essential for the hatching success in amphibians as seen in spotted salamander, *Ambystoma maculatum* , and the blue‐spotted salamander, *A. laterale* (Sacerdote and King 2009). As increases in salinity and temperature reduce dissolved oxygen levels (Jack and Khalifa 2009), tadpoles might have to adapt to strategies that enable their survival. In American Green treefrog, *Hyla cinerea* , locally adapted coastal populations showed higher survival, accelerated larval growth rates, and shorter larval periods than inland freshwater populations, regardless of salinity. This faster‐paced lifestyle is an adaptation to minimize larval mortality risk in highly variable and unpredictable saline environments. Mexican spadefoot toad, *Spea multiplicata* , demonstrates adaptive developmental plasticity by accelerating growth and undergoing earlier metamorphosis in response to drying conditions in ephemeral ponds (Burress et al. 2018). To navigate the trade‐off between delayed metamorphosis under osmotic stress (Gomez‐Mestre and Tejedo 2003) and accelerated growth due to oxygen stress, tadpoles might have developed local adaptations that enable them to strategically exploit such challenging yet unique habitats.

Selection of breeding site also appears to be contingent upon various factors such as distance of pools from the forest and the coastlines (Smith 1983; Egan et al. 2015). In this study, the distance of breeding pools from the forest varied between 0.1 and 74 m. Maximum tadpole occurrence was observed at the pools, which were at an average distance of 111.545 m from the coastline whereas the lowest abundance was observed at 41.187 m (Figure 4). As distance of pools from forest and coastline was negatively correlated, results suggest tadpole occurrence was higher in pools farther from the coastline and closer to the forest. Additionally, tadpoles were more likely to occur in shallow pools. This pattern may reflect the use of shallow pools in association with higher tadpole occurrence, as larger breeding pools also had greater depth (*r* = 0.27; *p* = 0.02) and may increase exposure to predators such as fishes and crabs (Gray and Christy 2000). On the other hand, shallow pools are also prone to drying leading to tadpole desiccation. This pattern suggests non‐random breeding site use, although adult oviposition choice was not directly tested, and such habitat use may be especially important for survival in dynamic and unpredictable coastal landscapes. The non‐linear and bimodal patterns observed for dissolved oxygen and distance from forest (Figure 4) likely reflect heterogeneous habitat conditions and trade‐offs among environmental factors such as salinity, pool depth, and distance from coastline, rather than simple linear preferences. Since environmental conditions in coastal pools fluctuate over time, our measurements provide a snapshot of habitat conditions during the survey period rather than a comprehensive characterization of conditions across the full breeding season. Furthermore, because our findings are based on tadpole occurrence rather than direct observations of egg deposition or larval survival, they should be interpreted as evidence of breeding site use rather than definitive adult oviposition preference.

Future research direction should build on both the literature synthesis and field results by moving beyond the current bias toward agricultural habitats, and explicitly examine coastal pools as breeding environments for *D. japonicus* . Integrating repeated surveys across the breeding season with direct observations of oviposition and measures of larval performance would help determine whether the patterns reported here reflect stable habitat use or short‐term occupancy under fluctuating coastal conditions. In addition, comparative studies across coastal and inland systems would clarify how environmental variability, particularly salinity and hydroperiod instability, shapes breeding‐site selection and reproductive success in this species.

## 5 Conclusion

This study provides new insight into breeding site use by *D. japonicus* in coastal landscapes, revealing how topographic context, pool structure, and water chemistry interact to shape tadpole occurrence patterns and may represent important environmental correlates of breeding site use. Our results highlight those coastal pools, particularly those occurring near natural vegetation and within specific topographic ranges, serve as critical breeding habitats on Jeju Island, where the basaltic substrate limits the formation of conventional inland freshwater habitats. Our results also suggest that *D. japonicus* may have more ecological flexibility than previously recognized, particularly in its ability to occur in moderately saline pools used for breeding. This raises new questions about how much salinity the species can tolerate, whether coastal populations show local adaptations, and how these factors influence tadpole survival, growth, and stress responses. Future studies should identify the salinity thresholds that limit successful reproduction and examine how breeding site use and oviposition patterns vary under changing coastal conditions.

## Author Contributions

**Kyongman Heo:** conceptualization (equal), data curation (lead), formal analysis (equal), funding acquisition (supporting), investigation (lead), methodology (lead), resources (equal), software (supporting), visualization (supporting), writing – original draft (equal), writing – review and editing (equal). **Deyatima Ghosh:** conceptualization (equal), data curation (supporting), formal analysis (lead), methodology (supporting), resources (equal), software (equal), supervision (supporting), validation (lead), visualization (supporting), writing – original draft (supporting), writing – review and editing (lead). **Kevin R. Messenger:** data curation (supporting), formal analysis (supporting), funding acquisition (supporting), investigation (supporting), project administration (lead), resources (supporting), supervision (lead), validation (supporting), writing – review and editing (supporting). **Siti N. Othman:** conceptualization (equal), data curation (supporting), formal analysis (supporting), funding acquisition (supporting), methodology (equal), software (equal), supervision (lead), visualization (lead), writing – original draft (supporting), writing – review and editing (equal), validation (lead).

## Funding

This work was supported by the Research Fund for International Scientists (RFIS) of the National Natural Science Foundation of China (NSFC; 32350410398), the Foreign Youth Talent Program of the Ministry of Science and Technology of the People's Republic of China (QN2023014005L), both awarded to SNO and Citizen Science Grants ‘Pul‐ssi’ 2020 from Korea Safety Health Environment Foundation and Donga Science to K.H.

## Ethics Statement

All quantitative field surveys were approved by the Nanjing Forestry University Institutional Animal Care and Use Committee (IACUC permit: 2023012), with no harm to tadpoles or egg clutches.

## Conflicts of Interest

The authors declare no conflicts of interest.

## Supporting information


**Table S1:** Publications dataset for meta‐analysis, synthesizing the ecological and breeding‐site characteristics of *Dryophytes japonicus* across East Asia.
**Table S2:** Field observation dataset including environmental and topographic variables measured in coastal breeding pools, and tadpole occurrence records.
**Table S3:** Shapiro–Wilk normality test results for environmental variables before and after log‐transformation.
**Table S4:** Frequency of publications across ecological themes and habitat types for *Dryophytes japonicus* .
**Table S5:** Frequency of publications across ecological themes and decade categories.
**Table S6:** Frequency of publications across habitat types and decade.
**Table S7:** Comparison of Akaike information criterion (AIC) among candidate GLMs used to model breeding microhabitat selection in *Dryophytes japonicus* .
**Table S8:** Summarized eigenvalues and variance explained by principal components.
**Table S9:** The loading values for each environmental and topographic variable on PC1–PC3 from the principal component analysis.
**Table S10:** The performance statistics of the Random Forest classification model used to predict tadpole occurrence.
**Table S11:** MeanDecreaseGini values for each environmental and topographic predictor used in the Random Forest classification model (Table S6; Figure 5).
**Figure S1:** Coastal breeding habitat of *Dryophytes japonicus* at Sae Island (Site B).
**Figure S2:** Correlation matrix of environmental variables and species occurrence.

## Data Availability

All data supporting the findings of this study are provided in the Appendix S1.
