# Supplementary material for: Ocean Romance: Japanese Treefrogs Exploit Coastal Pools for Breeding Under Salinity and Habitat Constraints
Source: Ecol Evol. 2026 May 28;16(6):e73744. doi: 10.1002/ece3.73744 (PMC13239210; doi:10.1002/ece3.73744)
Supplement: Supplementary file 1 — Table S1: Publications dataset for meta‐analysis, synthesizing the ecological and breeding‐site characteristics of Dryophytes japonicus across East Asia. Table S2: Field observation dataset including environmental and topographic variables measured in coastal breeding pools, and tadpole occurrence records. Table S3: Shapiro–Wilk normality test results for environmental variables before and after log‐transformation. Table S4: Frequency of publications across ecological themes and habitat types for Dryophytes japonicus . Table S5: Frequency of publications across ecological themes and decade categories. Table S6: Frequency of publications across habitat types and decade. Table S7: Comparison of Akaike information criterion (AIC) among candidate GLMs used to model breeding microhabitat selection in Dryophytes japonicus . Table S8: Summarized eigenvalues and variance explained by principal components. Table S9: The loading values for each environmental and topographic variable on PC1–PC3 from the principal component analysis. Table S10: The performance statistics of the Random Forest classification model used to predict tadpole occurrence. Table S11: MeanDecreaseGini values for each environmental and topographic predictor used in the Random Forest classification model (Table S6; Figure 5). Figure S1: Coastal breeding habitat of Dryophytes japonicus at Sae Island (Site B). Figure S2: Correlation matrix of environmental variables and species occurrence. [file ECE3-16-e73744-s001.docx]

**Appendix A**

**Supplementary Tables**

**Table S1.** **Publications dataset for meta-analysis, synthesizing the ecological and breeding-site characteristics of *Dryophytes japonicus* across East Asia.** All available studies that examined *D. japonicus* in relation to environmental or reproductive variables were included in the analysis, regardless of whether they provided quantitative data. All publications listed were used for the chi-square analysis.

| **Theme** | **Publication** | **Habitat type** | **Year** | **Author** |
| --- | --- | --- | --- | --- |
| Breeding | Breeding preferences in the treefrogs *Dryophytes japonicus* (Hylidae) in Mongolia | Natural pool | 2019 | Borzée, A., Purevdorj, Z., Kim, Y. I., Kong, S., Choe, M., Yi, Y., ... & Jang, Y. |
|  | Seasonal Pattern of Advertisement Calling and Physiology in Prolonged Breeding Anurans, Japanese Tree Frog (*Dryophytes japonicus*) | Agricultural land | 2023 | Park, J. K., & Do, Y. |
|  | Breeding range variation between Korean hylids (*Dryophytes* sp.) | Agricultural land | 2019 | Kim, E., Cahyana, A. N., Jang, Y., & Borzée, A. |
|  | Relationship between agro-environmental variables and breeding Hylids in rice paddies | Agricultural land | 2018 | Borzée, A., Heo, K., & Jang, Y. |
|  | Large-Scale Hybridisation as an Extinction Threat to the Suweon Treefrog (Hylidae: *Dryophytes suweonensis*) | Agricultural land | 2020 | Borzée, A., Fong, J. J., Nguyen, H. Q., & Jang, Y. |
|  | Breeding phenology and landscape use in all amphibian species from the Republic of Korea based on open-source data | Controlled condition | 2022 | Groffen, J., Andersen, D., & Borzée, A. |
|  | Evaluation of Deep Learning-Based Monitoring of Frog Reproductive Phenology | Controlled condition | 2023 | Kimura, K., & Sota, T. |
|  | A study on the breeding habits of *Hyla arborea* *japonica* Guenther | Agricultural land | 1931 | INUKAI, T., & OCHIAI, S. |
|  | Differential responses of two anuran species breeding in rice fields to landscape composition and spatial scale | Agricultural land | 2010 | Kato, N., Yoshio, M., Kobayashi, R., & Miyashita, T. |
|  | Plasticity of mating calls in hyla japonica (Amphibia: Hylidae) | Agricultural land | 1998 | Park, K. B., & Choe, J. C. |
|  | No reproductive character displacement in male advertisement signals of *Hyla japonica* in relation to the sympatric *H. suweonensis* | Agricultural land | 2013 | Park, S., Jeong, G., & Jang, Y. |
| Ecology | Asymmetric competition over calling sites in two closely related treefrog species | Agricultural land | 2016 | Borzée, A., Kim, J. Y., & Jang, Y. |
|  | Relationship between anuran larvae occurrence and aquatic environment in septentrional east Palearctic landscapes | Natural pool | 2021 | Borzée, A., Kim, Y. I., Purevdorj, Z., Maslova, I., Schepina, N., & Jang, Y. |
|  | Preference for natural borders in rice paddies by two treefrog species | Agricultural land | 2018 | Groffen, J., Borzée, A., & Jang, Y. |
|  | Effects of landscape heterogeneity at multiple spatial scales on paddy field-breeding frogs in a large alluvial plain in Japan | Agricultural land | 2022 | Matsushima, N., Hasegawa, M., & Nishihiro, J. |
|  | Survey of the distribution of various frog species in each paddy field, focusing on spatial autocorrelation | Agricultural land | 2023 | Niwa, H., & Uno, Y. |
|  | East palearctic treefrog past and present habitat suitability using ecological niche models | Controlled condition | 2022 | Andersen, D., Maslova, I., Purevdorj, Z., Li, J. T., Messenger, K. R., Ren, J. L., ... & Borzée, A. |
|  | Spring voices in Korean rice fields: The effect of abiotic variables and syntopic calls on the calling activity of the treefrog Dryophytes suweonensis | Agricultural land | 2020 | Amael, B., Eunchong, S., Sunmin, O., & Yikweon, J. |
|  | Multi-Spatial-Scale Factors Determining the Abundance of Frogs in Rice Paddy Fields and Their Potential as Biological Control Agents | Agricultural land | 2023 | Baba, Y. G., Osawa, T., Kusumoto, Y., & Tanaka, K. |
|  | Relationship between body size variation and habitat environment of Hyla japonica in Jeju Island, south Korea | Natural pool | 2018 | Koo, K. S., Kwon, S., Park, I. K., & Oh, H. S. |
|  | Spatiotemporal distributions of the endangered treefrog, Hyla suweonensis, in relation to sympatric H. japonica: calling activity and habitat characteristics | Agricultural land | 2013 | ROH, G., & JANG, Y. |
|  | Effects of habitat environment and land use on the abundance of Japanese tree frog (*Hyla japonica*) in Incheon, Korea | Agricultural land | 2017 | Park, S. H., Cho, H., Jin, S. N., & Cho, K. H. |
|  | Relationship among call variables and environmental factors, call property differences between Bd infected and non-infected groups, and territoriality in Hyla japonica | Agricultural land | 2014 | An, D. |
|  | Negative effects of deep roadside ditches on *Pelophylax porosa brevipoda* dispersal and migration in comparison with Hyla japonica in a rice paddy area in Japan | Agricultural land | 2012 | Naito, R., Sakai, M., & Morimoto, Y. |
|  | Abiotic effects on calling phenology of three frog species in Korea | Artificial pond | 2012 | Yoo, E., & Jang, Y. |
|  | Dynamical Calling Behavior Experimentally Observed in Japanese Tree Frogs (*Hyla japonica*) | Agricultural land | 2007 | Aihara, I., Horai, S., Kitahata, H., Aihara, K., & Yoshikawa, K. |
|  | Individual and sexual differences in time to habituate to food-stimuli presentation of potential prey in *Hyla japonica* | Artificial pond | 2019 | Tanabe, S., Kasuya, E., & Miyatake, T. |
|  | The Japanese tree frog (*Hyla japonica*), one of the most cold-resistant species of amphibians | Natural pool | 2016 | Berman, D. I., Meshcheryakova, E. N., & Bulakhova, N. A. |
|  | Comparison of health status of Japanese tree frog (Hyla Japonica) in a rural and an urban area | Agricultural land | 2017 | Park, S. H., & Cho, K. H. |
|  | Intraspecific competition reduces the quantity of excreted nutrients in tadpoles | Agricultural land | 2020 | Ramamonjisoa, N., Rakotonoely, H., Kwon, T., Nakanishi, K., & Natuhara, Y. |
|  | Periodic rhythm and anti-phase synchronization in calling behaviors of Japanese rain frogs | Agricultural land | 2006 | Ikkyu Aihara, H. Kitahata, K. Aihara, K. Yoshikawa |
|  | Enhanced call effort in Japanese tree frogs infected by amphibian chytrid fungus | Agricultural land | 2016 | An, D., & Waldman, B. |
|  | Call Alternation Between Specific Pairs of Male Frogs Revealed by a Sound-Imaging Method in Their Natural Habitat. | Natural pool | 2016 | Ikkyu Aihara1, Takeshi Mizumoto2, Hiromitsu Awano3, Hiroshi G. Okuno |
|  | Geographic variation in advertisement calls in a tree frog species: gene flow and selection hypotheses | Natural pool | 2011 | Jang, Y., Hahm, E. H., Lee, H. J., Park, S., Won, Y. J., & Choe, J. C. |
|  | Feeding habits of the Japanese tree frog, *Hyla japonica*, in the reproductive season | Agricultural land | 2000 | Hirai, T., & Matsui, M. |
|  | Feeding relationships between *Hyla japonica* and *Rana nigromaculata* in rice fields of Japan | Agricultural land | 2002 | Hirai, T., & Matsui, M. |
|  | Diet composition of Japanese tree frog (*Hyla japonica*) in a rice paddy, South Korea | Agricultural land | 2018 | Park, S. H., Lee, H., & Cho, K. H. |
|  | A note on prey composition of the Japanese treefrog, Hyla japonica, in an area invaded by Argentine ants, Linepithema humile | Artificial pond | 2009 | Ito, F., Okaue, M., & Ichikawa, T. |
|  | Defensive function of petiole spines in queens and workers of the formicine ant Polyrhachis lamellidens (Hymenoptera: Formicidae) against an ant predator, the Japanese treefrog *Hyla japonica* | Agricultural land | 2016 | Ito, F., Taniguchi, K., & Billen, J. |
|  | Temporal variation in behavioral responses to dietary cues from a gape‐limited predator in tadpole prey: A test of the phylogenetic relatedness hypothesis | Agricultural land | 2019 | Ramamonjisoa, N., & Mori, A. |
|  | Diet composition of *Hyla japonica* in soybean fields: is a euryphagous predator useful for pest management? | Agricultural land | 2007 | Hirai, T. |
|  | Morphology, Age Structure and Mating Call Characteristics of Japanese tree frog (*Hyla Japonica*) and Suweon tree frog (*Hyla Suweonensis*) | Agricultural land | 2014 | Park S., & Cho G. |
|  | Comparison of the abundance, morphology and diets of Japanese tree frogs (*Hyla japonica*) living in rural and urban areas | Agricultural land | 2019 | Park S., & Cho G. |
|  | Comparison study about snout-vent length and biomass of the endangered species II, Narrow-mouth Toad (*Kaloula borealis*), and other amphibians (Salientia: *Bombina orientalis, Hyla japonica, Rana nigromaculata*) in some areas of South Korea | Agricultural land | 2017 | An, C |
|  | Study on feeding preference of order Salientia (Amphibia) in Korea | Agricultural land | 2016 | Joo, G. |
|  | Distribution Characteristics and Body Size Variation of Japanese Tree Frog (*Hyla japonica*) in South Korea | Controlled condition | 2019 | Koo, K. |
| Hibernation | Microhabitat use during brumation in the Japanese treefrog, *Dryophytes japonicus* | Agricultural land | 2018 | Borzée, A., Kim, M., Kim, J. Y., Kim, T., & Jang, Y. |
|  | Interspecific variation in seasonal migration and brumation behavior in two closely related species of treefrogs | Agricultural land | 2019 | Borzee, A., Choi, Y., Kim, Y. E., Jablonski, P. G., & Jang, Y. |
|  | Interference competition driven by hydric stress in Korean Hylids | Agricultural land | 2018 | Amaël, B., & Yikweon, J. |
|  | Involvement of glucose in freeze tolerance in the Japanese tree frog *Hyla japonica* | Agricultural land | 2022 | Okada, R., Adachi, S., Takiya, Y., Iwasaki, R., Hirota, A., & Kikuyama, S. |
|  | Molecular cloning of cDNA encoding an aquaglyceroporin, AQP-h9, in the Japanese tree frog, *Hyla japonica*: possible roles of AQP-h9 in freeze tolerance | Controlled condition | 2015 | Hirota, A., Takiya, Y., Sakamoto, J., Shiojiri, N., Suzuki, M., Tanaka, S., & Okada, R. |
|  | Cold stress and light signals induce the expression of cold-inducible RNA binding protein (cirp) in the brain and eye of the Japanese treefrog (*Hyla japonica*) | Controlled condition | 2008 | Kenkichi Sugimoto 1, Huijie Jiang |
| Pathology | High ranavirus infection rates at low and extreme temperatures in the tadpoles of Japanese treefrogs (*Dryophytes japonicus*) that breed in rice paddies in the summer | Agricultural land | 2023 | Roh, N. H., Kim, J., Park, J., & Park, D. |
|  | Introduced bullfrogs are associated with increased Batrachochytrium dendrobatidis prevalence and reduced occurrence of Korean treefrogs | Agricultural land | 2017 | Borzée, A., Kosch, T. A., Kim, M., & Jang, Y. |
|  | Prevalence of Ranavirus infection in three anuran species across South Korea | Agricultural land | 2022 | Roh, N., Park, J., Kim, J., Kwon, H., & Park, D. |
|  | PCR detection of ranavirus from dead *Kaloula borealis* and sick *Hyla japonica* tadpoles in the wild | Agricultural land | 2017 | Park, I. K., Koo, K. S., Moon, K. Y., Lee, J. G., & Park, D. |
| Morphology | Colour variants in the Japanese Treefrog (*Dryophytes japonicus*) from Russia and Korea. | Agricultural land | 2018 | Maslova, I., Jang, Y., Zhestkov, A., & Borzée, A. |
|  | Testing multiple hypotheses on the colour change of treefrogs in response to various external conditions | Agricultural land | 2023 | Park, C., No, S., Yoo, S., Oh, D., Hwang, Y., Kim, Y., & Kang, C. |
|  | Genetic and morphologic studies on ten albino stocks in *Hyla arborea japonica* | Agricultural land | 1977 | Nishioka, M., & Ueda, H. |
|  | Colour and pattern change against visually heterogeneous backgrounds in the tree frog *Hyla japonica* | Agricultural land | 2016 | Kang, C., Kim, Y. E., & Jang, Y. |
|  | Morphological characterization and classification of anuran tadpoles in Korea | Agricultural land | 2006 | Park, D. S., Cheong, S. K., & Sung, H. C. |
|  | Predator-specific inducible morphological changes in two Japanese anuran tadpoles | Agricultural land | 2013 | Takahara, T., Kohmatsu, Y., Maruyama, A., & Yamaoka, R. |
|  | Morphometrics of two sympatric species of tree frogs in Korea: a morphological key for the critically endangered *Hyla suweonensis* in relation to *H. japonica* | Agricultural land | 2013 | Borzée, A., Park, S., Kim, A., Kim, H. T., & Jang, Y. |
|  | Background matching by means of dorsal color change in treefrog populations (*Hyla japonica*) | Agricultural land | 2014 | Choi, N., & Jang, Y. |
|  | Comparative skeletogenesis of the oriental tree frog *Hyla orientalis* (Anura: Hylidae) | Agricultural land | 2014 | Choi, N., & Jang, Y. |
| Behavior | First record of heterospecific amplexus behaviour between *Pelophylax chosenicus* (Anura: Ranidae) and *Dryophytes japonicus* (Anura: Hylidae) in Paju, Republic of Korea | Agricultural land | 2021 | Koo, K. S., Kwon, S., Kwak, M., & Oh, J. |
|  | When Fleeing Matters: Differences in Escape Behaviours of Three Northeast Asian Anurans | Natural pool | 2023 | YI, Y., PUREVDORJ, Z., MASLOVA, I., JANG, Y., & BORZÉE, A. |
|  | Variations in boldness, behavioural and physiological traits of an endangered and a common hylid species from Korea | Agricultural land | 2018 | Borzée, A., Yu, A. Y., & Jang, Y. |
|  | Strategy of landing behavior of the tree frog *Hyla japonica* | Controlled condition | 2017 | Kamada, K., Tachibanagi, R., & Nakagawa, H. |
|  | Modeling synchronized calling behavior of Japanese tree frogs | Controlled condition | 2009 | Aihara, I. |
|  | Benefit of suites of defensive behavior induced by predator chemical cues on anuran tadpoles, *Hyla japonica* | Agricultural land | 2008 | Takahara, T., Kohmatsu, Y., Maruyama, A., & Yamaoka, R. |
|  | Behavior of Japanese tree frogs under microgravity on MIR and in parabolic flight | Controlled condition | 1994 | Izumi-Kurotani, A., Yamashita, M., Kawasaki, Y., Kurotani, T., Mogami, Y., Okuno, M., ... & Naitoh, T. |
|  | Predator-avoidance behavior in anuran tadpoles: a new bioassay for characterization of water-soluble cues | Agricultural land | 2008 | Takahara, T., Kohmatsu, Y., & Yamaoka, R. |
|  | Inducible defense behavior of an anuran tadpole: cue-detection range and cue types used against predator | Agricultural land | 2012 | Takahara, T., Kohmatsu, Y., Maruyama, A., Doi, H., Yamanaka, H., & Yamaoka, R. |
|  | Energy efficient self-organizing control for wireless sensor networks inspired by calling behavior of frogs | Agricultural land | 2012 | Takahara, T., Kohmatsu, Y., Maruyama, A., Doi, H., Yamanaka, H., & Yamaoka, R. |
| Predation | Feeding habits of the endangered Japanese diving beetle Hydaticus bowringii (Coleoptera: Dytiscidae) larvae in paddy fi elds and implications for its conservation. | Agricultural land | 2020 | Watanabe, R., Ohba, S. Y., & Yokoi, T. |
|  | The invasive American bullfrog (*Lithobates catesbeianus*) in the Republic of Korea: history and recommendations for population control. | Agricultural land | 2019 | Groffen, J., Kong, S., Jang, Y., & Borzée, A. |
|  | Metabarcoding of feces and intestinal contents to determine carnivorous diets in red-crowned cranes in eastern Hokkaido, Japan | Controlled condition | 2022 | Kataoka, H., Koita, N., Kondo, N. I., Ito, H. C., Nakajima, M., Momose, K., ... & Teraoka, H. |
|  | First amphibian behavioural observation from the Democratic People’s Republic of Korea: predation of a Dryophytes japonicus tadpole by *Hydaticus* sp. Larvae | Natural pool | 2019 | Borzée, А. |
|  | Field observations of predatory behavior by juvenile rice frogs (*Fejervarya kawamurai*) on Japanese tree frogs (*Hyla japonica*) | Agricultural land | 2014 | Doi, T. |
|  | Frog- and lizard-eating behaviour of wild Japanese macaques in Yakushima, Japan | Natural pool | 1990 | Suzuki, S., Hill, D. A., Maruhashi, T., & Tsukahara, T. |
|  | Toward an understanding of tree frog (*Hyla japonica*) for predator deterrence | Controlled condition | 2021 | Chai, L., Yin, C., Kamau, P. M., Luo, L., Yang, S., Lu, X., ... & Wang, Y. |
|  | Anuran-dependent predation by the giant water bug, Lethocerus deyrollei (Hemiptera: Belostomatidae), in rice fields of Japan | Agricultural land | 2002 | Hirai, T., & Hidaka, K. |
|  | Different chemical cues originating from a shared predator induce common defense responses in two prey species | Agricultural land | 2013 | Takahara, T., Doi, H., Kohmatsu, Y., & Yamaoka, R. |
|  | Specific behavioral responses of *Hyla japonica* tadpoles to chemical cues released by two predator species | Agricultural land | 2006 | Takahara, T., Kohmatsu, Y., Maruyama, A., & Yamaoka, R. |
|  | Foods Use of the Red-Tongued Viper Snake (*Gloydius ussuriensis*) | Natural pool | 2014 | Kim, B., & Oh, H. |
| Conservation | Influences of water control and application of rice bran on density of Japanese tree frog, *Hyla japonica*, larvae and spiders in paddy field. | Agricultural land | 2004 | Oyama, J., & Kidokoro, T. |
|  | Assessing the impact of large-scale farmland abandonment on the habitat distributions of frog species after the Fukushima nuclear accident | Agricultural land | 2021 | Matsushima, N., Ihara, S., Inaba, O., & Horiguchi, T. |
|  | Seoul, keep your paddies! Implications for the conservation of hylid species | Agricultural land | 2015 | Borzée, A., Ahn, J., Kim, S., Heo, K., & Jang, Y. |
|  | Paddy-associated frog declines via urbanization: a test of the dispersal-dependent-decline hypothesis | Agricultural land | 2011 | Tsuji, M., Ushimaru, A., Osawa, T., & Mitsuhashi, H. |
|  | Systematic implications of hybridization experiments with some Eurasian treefrogs (Genus *Hyla*) | Controlled condition | 1984 | Kuramoto, M. |
|  | Indirect positive effects of agricultural modernization on the abundance of Japanese tree frog tadpoles in rice fields through the release from predators | Agricultural land | 2013 | Katayama, N., Goto, T., Narushima, F., Amano, T., Kobori, H., & Miyashita, T. |
| Water quality and salinity | Impact of Water Quality on the Occurrence of Two Endangered Korean Anurans: *Dryophytes suweonensis* and *Pelophylax chosenicus* | Agricultural land | 2018 | Borzée, A., Kyong, C. N., Kil, H. K., & Jang, Y. |
|  | First report of *Dryophytes japonicus* tadpoles in saline environment. | Natural pool | 2019 | Heo, K., Kim, Y. I., Bae, Y., Jang, Y., & Borzee, A. |
|  | Effects of water management, connectivity, and surrounding land use on habitat use by frogs in rice paddies in Japan | Agricultural land | 2012 | Naito, R., Yamasaki, M., Natuhara, Y., & Morimoto, Y. |

**Table S2. Field observation dataset including environmental and topographic variables measured in coastal breeding pools, and tadpole occurrence records.** This dataset includes population/site identity, sample ID, sampling date, species, locality, and geographic coordinates (latitude and longitude). For each coastal pool, habitat type and measured environmental variables are provided: surface area (Area/ m²), salinity (sal/ ppt), water temperature (temp/ °C), dissolved oxygen (DO/ mg/L), distance from coastline (DtC/ m), distance from forest (DtF /m), pool depth (m), and elevation (m). Tadpole presence or absence is reported under ‘Occurrence’.

| **Population/**  **site** | **Sample ID** | **Species** | **Locality** | **Latitude** | **Longitude** | **Habitat** | **Area** | **Sal** | **Temp** | **DO** | **DtC** | **DtF** | **Depth** | **Occurence** | **Elevation** |
| --- | --- | --- | --- | --- | --- | --- | --- | --- | --- | --- | --- | --- | --- | --- | --- |
| A | DJJG001 | *Dryophytes japonicus* | Seoguipo, Jeju | 33.2328 | 126.4888 | Brackish pool | 1.204 | 0.4 | 29.4 | 12.7 | 238 | 5.371 | 0.31 | 40 | 15 |
| A | DJJG002 | *Dryophytes japonicus* | Seoguipo, Jeju | 33.2327 | 126.4889 | Brackish pool | 0.181 | 0.1 | 28 | 13.4 | 231 | 5.076 | 0.278 | 5 | 14 |
| A | DJJG003 | *Dryophytes japonicus* | Seoguipo, Jeju | 33.2326 | 126.4891 | Brackish pool | 0.82 | 0.1 | 31.2 | 11.7 | 212 | 4.184 | 0.307 | 15 | 12 |
| A | DJJG004 | *Dryophytes japonicus* | Seoguipo, Jeju | 33.2321 | 126.4905 | Brackish pool | 1.109 | 0.1 | 31.8 | 11.3 | 209 | 4.649 | 0.337 | 50 | 6 |
| A | DJJG005 | *Dryophytes japonicus* | Seoguipo, Jeju | 33.2321 | 126.4903 | Brackish pool | 0.628 | 0.1 | 31 | 11 | 59 | 11.274 | 0.293 | 10 | 6 |
| A | DJJG006 | *Dryophytes japonicus* | Seoguipo, Jeju | 33.2324 | 126.4903 | Brackish pool | 1.824 | 0.1 | 29.4 | 12.4 | 85 | 1.76 | 0.339 | 10 | 8 |
| A | DJJG007 | *Dryophytes japonicus* | Seoguipo, Jeju | 33.2324 | 126.4901 | Brackish pool | 0.196 | 0.2 | 31.1 | 11.8 | 103 | 7.04 | 0.482 | 10 | 8 |
| A | DJJG008 | *Dryophytes japonicus* | Seoguipo, Jeju | 33.23251 | 126.4902 | Brackish pool | 0.136 | 1.2 | 28.6 | 11.6 | 106 | 2.95 | 0.229 | 10 | 9 |
| A | DJJG009 | *Dryophytes japonicus* | Seoguipo, Jeju | 33.2324 | 126.4903 | Brackish pool | 0.076 | 1.5 | 30.8 | 12.8 | 86 | 6.91 | 0.304 | 5 | 8 |
| A | DJJG010 | *Dryophytes japonicus* | Seoguipo, Jeju | 33.23228 | 126.4904 | Brackish pool | 0.887 | 0.3 | 30.4 | 12.8 | 74 | 13.21 | 0.411 | 0 | 7 |
| A | DJJG011 | *Dryophytes japonicus* | Seoguipo, Jeju | 33.23229 | 126.4906 | Brackish pool | 6.096 | 0.1 | 28.3 | 11.6 | 58 | 3.22 | 0.279 | 10 | 6 |
| A | DJJG012 | *Dryophytes japonicus* | Seoguipo, Jeju | 33.23251 | 126.4903 | Brackish pool | 0.171 | 0.1 | 29.5 | 11.5 | 92 | 6.4 | 0.228 | 5 | 8 |
| B | DJJS001 | *Dryophytes japonicus* | Seoguipo, Jeju | 33.23612 | 126.5607 | Brackish pool | 2.584 | 0.4 | 29.7 | 13 | 24 | 24 | 0.183 | 5 | 9 |
| B | DJJS002 | *Dryophytes japonicus* | Seoguipo, Jeju | 33.2361 | 126.5607 | Brackish pool | 4.231 | 0.3 | 29.9 | 13.3 | 38 | 19 | 0.22 | 30 | 9 |
| B | DJJS003 | *Dryophytes japonicus* | Seoguipo, Jeju | 33.23607 | 126.5607 | Brackish pool | 3.091 | 0.3 | 30.8 | 12.6 | 45 | 17 | 0.353 | 30 | 9 |
| B | DJJS004 | *Dryophytes japonicus* | Seoguipo, Jeju | 33.23607 | 126.5608 | Brackish pool | 1.143 | 0.4 | 30.5 | 13.1 | 40 | 20 | 0.212 | 10 | 9 |
| B | DJJS005 | *Dryophytes japonicus* | Seoguipo, Jeju | 33.23605 | 126.5608 | Brackish pool | 0.565 | 0.6 | 29.3 | 14.2 | 43 | 23 | 0.201 | 30 | 9 |
| B | DJJS006 | *Dryophytes japonicus* | Seoguipo, Jeju | 33.23611 | 126.5608 | Brackish pool | 0.964 | 0.5 | 30.1 | 13.4 | 42 | 16 | 0.221 | 20 | 10 |
| B | DJJS007 | *Dryophytes japonicus* | Seoguipo, Jeju | 33.23607 | 126.5608 | Brackish pool | 0.317 | 0.3 | 30.7 | 11.5 | 48 | 18 | 0.285 | 10 | 10 |
| B | DJJS008 | *Dryophytes japonicus* | Seoguipo, Jeju | 33.23606 | 126.5609 | Brackish pool | 1.022 | 0.4 | 28.3 | 14 | 42 | 7 | 0.224 | 5 | 10 |
| B | DJJS009 | *Dryophytes japonicus* | Seoguipo, Jeju | 33.23605 | 126.5609 | Brackish pool | 0.172 | 0.2 | 28.5 | 13.5 | 43 | 22 | 0.105 | 10 | 10 |
| B | DJJS010 | *Dryophytes japonicus* | Seoguipo, Jeju | 33.23603 | 126.5609 | Brackish pool | 0.16 | 0.1 | 29.8 | 11.7 | 33 | 31 | 0.425 | 0 | 10 |
| B | DJJS011 | *Dryophytes japonicus* | Seoguipo, Jeju | 33.23603 | 126.5609 | Brackish pool | 0.04 | 1.8 | 29.4 | 12.4 | 44 | 30 | 0.27 | 10 | 10 |
| B | DJJS012 | *Dryophytes japonicus* | Seoguipo, Jeju | 33.23598 | 126.561 | Brackish pool | 6.597 | 0.4 | 29.9 | 13.6 | 48 | 5 | 0.3 | 5 | 10 |
| B | DJJS013 | *Dryophytes japonicus* | Seoguipo, Jeju | 33.23596 | 126.5609 | Brackish pool | 8.796 | 0.5 | 29.8 | 13.9 | 54 | 16 | 0.224 | 10 | 9 |
| B | DJJS014 | *Dryophytes japonicus* | Seoguipo, Jeju | 33.23647 | 126.5603 | Brackish pool | 911.42 | 0.7 | 31.2 | 13.4 | 51.02 | 7.23 | 0.651 | 0 | 8 |
| B | DJJS015 | *Dryophytes japonicus* | Seoguipo, Jeju | 33.2367 | 126.5597 | Brackish pool | 484.026 | 0.1 | 29.4 | 12.3 | 30.7 | 31.31 | 0.214 | 0 | 4 |
| B | DJJS016 | *Dryophytes japonicus* | Seoguipo, Jeju | 33.23671 | 126.5603 | Brackish pool | 119.004 | 0.8 | 29.7 | 12.1 | 26.54 | 49.27 | 0.152 | 0 | 8 |
| B | DJJS017 | *Dryophytes japonicus* | Seoguipo, Jeju | 33.2357 | 126.5608 | Brackish pool | 0.204 | 19.8 | 30.2 | 13.8 | 12.97 | 62.97 | 0.199 | 0 | 7 |
| B | DJJS018 | *Dryophytes japonicus* | Seoguipo, Jeju | 33.2357 | 126.5608 | Brackish pool | 0.172 | 25.4 | 29.5 | 11.4 | 7.59 | 74.25 | 0.295 | 0 | 7 |
| B | DJJS019 | *Dryophytes japonicus* | Seoguipo, Jeju | 33.23559 | 126.5615 | Brackish pool | 0.456 | 8.9 | 30.3 | 13.6 | 10.23 | 71.58 | 0.432 | 0 | 8 |
| B | DJJS020 | *Dryophytes japonicus* | Seoguipo, Jeju | 33.23566 | 126.5606 | Brackish pool | 0.262 | 2.3 | 28.2 | 11.2 | 12.28 | 66.54 | 0.105 | 10 | 6 |
| B | DJJS021 | *Dryophytes japonicus* | Seoguipo, Jeju | 33.23565 | 126.5607 | Brackish pool | 0.241 | 0.7 | 28.4 | 11.3 | 21 | 59.25 | 0.425 | 10 | 6 |
| B | DJJS022 | *Dryophytes japonicus* | Seoguipo, Jeju | 33.23562 | 126.5607 | Brackish pool | 0.342 | 0.5 | 29.1 | 11.1 | 26 | 60.22 | 0.27 | 5 | 6 |
| B | DJJS023 | *Dryophytes japonicus* | Seoguipo, Jeju | 33.23592 | 126.5606 | Brackish pool | 0.425 | 0.3 | 30.7 | 11.3 | 24 | 59.24 | 0.444 | 5 | 8 |
| B | DJJS024 | *Dryophytes japonicus* | Seoguipo, Jeju | 33.23597 | 126.5607 | Brackish pool | 0.294 | 0.9 | 31.1 | 11.5 | 27 | 60.24 | 0.381 | 5 | 9 |
| B | DJJS025 | *Dryophytes japonicus* | Seoguipo, Jeju | 33.23601 | 126.5607 | Brackish pool | 0.601 | 0.6 | 29.9 | 11.2 | 24 | 54.95 | 0.236 | 5 | 8 |
| B | DJJS026 | *Dryophytes japonicus* | Seoguipo, Jeju | 33.23619 | 126.5599 | Brackish pool | 0.165 | 0.9 | 30 | 12.1 | 22 | 52.53 | 0.124 | 5 | 6 |
| B | DJJS027 | *Dryophytes japonicus* | Seoguipo, Jeju | 33.23583 | 126.5611 | Brackish pool | 0.224 | 0.8 | 28.8 | 11.9 | 27 | 55.29 | 0.251 | 0 | 9 |
| B | DJJS028 | *Dryophytes japonicus* | Seoguipo, Jeju | 33.23601 | 126.5611 | Brackish pool | 1.349 | 0.2 | 29.3 | 12.8 | 74.25 | 3 | 0.21 | 0 | 10 |
| B | DJJS029 | *Dryophytes japonicus* | Seoguipo, Jeju | 33.23615 | 126.5609 | Brackish pool | 0.966 | 0.3 | 29.5 | 11.1 | 75.32 | 2.54 | 0.302 | 0 | 11 |
| B | DJJS030 | *Dryophytes japonicus* | Seoguipo, Jeju | 33.23607 | 126.561 | Brackish pool | 0.501 | 0.1 | 30.3 | 12.9 | 77.24 | 4.64 | 0.165 | 0 | 11 |
| B | DJJS031 | *Dryophytes japonicus* | Seoguipo, Jeju | 33.23599 | 126.5611 | Brackish pool | 0.343 | 0.1 | 28.2 | 13.3 | 73.54 | 7 | 0.209 | 0 | 10 |
| B | DJJS032 | *Dryophytes japonicus* | Seoguipo, Jeju | 33.23544 | 126.5619 | Brackish pool | 0.549 | 0.1 | 29.6 | 11.4 | 46.14 | 28.53 | 0.286 | 0 | 8 |
| B | DJJS033 | *Dryophytes japonicus* | Seoguipo, Jeju | 33.23526 | 126.5625 | Brackish pool | 0.277 | 0.1 | 29.9 | 13.3 | 67.7 | 26.8 | 0.258 | 0 | 7 |
| B | DJJS034 | *Dryophytes japonicus* | Seoguipo, Jeju | 33.23557 | 126.563 | Brackish pool | 0.195 | 0.1 | 29.9 | 12.5 | 70.32 | 20.28 | 0.428 | 0 | 10 |
| B | DJJS035 | *Dryophytes japonicus* | Seoguipo, Jeju | 33.23515 | 126.5619 | Brackish pool | 0.506 | 0.5 | 30.2 | 11.9 | 29.51 | 62.9 | 0.32 | 0 | 5 |
| B | DJJS036 | *Dryophytes japonicus* | Seoguipo, Jeju | 33.23586 | 126.5609 | Brackish pool | 0.112 | 0.9 | 30.2 | 10.8 | 12.63 | 52.15 | 0.227 | 30 | 9 |
| B | DJJS037 | *Dryophytes japonicus* | Seoguipo, Jeju | 33.23706 | 126.5598 | Brackish pool | 0.422 | 0.6 | 28.8 | 13.5 | 14.8 | 50.12 | 0.144 | 30 | 4 |
| B | DJJS038 | *Dryophytes japonicus* | Seoguipo, Jeju | 33.23591 | 126.5604 | Brackish pool | 2.052 | 0.8 | 29.5 | 11.2 | 26.4 | 40.98 | 0.158 | 20 | 7 |
| B | DJJS039 | *Dryophytes japonicus* | Seoguipo, Jeju | 33.23604 | 126.5603 | Brackish pool | 0.39 | 5.8 | 27.7 | 14.8 | 30.1 | 36.19 | 0.419 | 20 | 7 |
| B | DJJS040 | *Dryophytes japonicus* | Seoguipo, Jeju | 33.23594 | 126.5602 | Brackish pool | 0.173 | 6.9 | 28.7 | 12 | 15.55 | 57.83 | 0.255 | 20 | 6 |
| B | DJJS041 | *Dryophytes japonicus* | Seoguipo, Jeju | 33.23585 | 126.5605 | Brackish pool | 0.264 | 2.1 | 29.7 | 12.2 | 18.2 | 49.83 | 0.225 | 40 | 7 |
| B | DJJS042 | *Dryophytes japonicus* | Seoguipo, Jeju | 33.23589 | 126.5603 | Brackish pool | 0.401 | 1.7 | 30.4 | 13 | 14.09 | 55.7 | 0.297 | 50 | 6 |
| B | DJJS043 | *Dryophytes japonicus* | Seoguipo, Jeju | 33.23623 | 126.5606 | Brackish pool | 0.259 | 0.3 | 30.2 | 11.6 | 34 | 43 | 0.212 | 10 | 9 |
| B | DJJS044 | *Dryophytes japonicus* | Seoguipo, Jeju | 33.23622 | 126.5606 | Brackish pool | 0.284 | 0.1 | 30.7 | 11.6 | 22 | 54 | 0.244 | 15 | 9 |
| B | DJJS045 | *Dryophytes japonicus* | Seoguipo, Jeju | 33.23618 | 126.5605 | Brackish pool | 0.401 | 0.2 | 31.6 | 12.6 | 36 | 54 | 0.358 | 30 | 9 |
| B | DJJS046 | *Dryophytes japonicus* | Seoguipo, Jeju | 33.23622 | 126.5606 | Brackish pool | 0.989 | 0.2 | 32.1 | 11.2 | 18 | 52 | 0.331 | 0 | 10 |
| B | DJJS047 | *Dryophytes japonicus* | Seoguipo, Jeju | 33.23624 | 126.5607 | Brackish pool | 0.612 | 0.2 | 32.4 | 12.8 | 17 | 48 | 0.137 | 0 | 10 |
| B | DJJS048 | *Dryophytes japonicus* | Seoguipo, Jeju | 33.23619 | 126.5606 | Brackish pool | 4.039 | 0.2 | 28.9 | 13.4 | 69 | 0.3 | 0.314 | 0 | 9 |
| B | DJJS049 | *Dryophytes japonicus* | Seoguipo, Jeju | 33.23618 | 126.5606 | Brackish pool | 2.191 | 0.1 | 28.8 | 13.4 | 70 | 0.452 | 0.351 | 0 | 9 |
| B | DJJS050 | *Dryophytes japonicus* | Seoguipo, Jeju | 33.23613 | 126.5606 | Brackish pool | 13.56 | 0.2 | 29.6 | 13 | 74 | 8 | 0.8 | 0 | 9 |
| B | DJJS051 | *Dryophytes japonicus* | Seoguipo, Jeju | 33.23605 | 126.5605 | Brackish pool | 7.805 | 0.4 | 29.3 | 14 | 59 | 13 | 0.36 | 0 | 8 |
| C | DJJO001 | *Dryophytes japonicus* | Seoguipo, Jeju | 33.23819 | 126.5479 | Brackish pool | 0.741 | 0.2 | 28.1 | 13.8 | 4.45 | 21.93 | 0.223 | 20 | 8 |
| C | DJJO002 | *Dryophytes japonicus* | Seoguipo, Jeju | 33.23813 | 126.5479 | Brackish pool | 0.214 | 0.4 | 28.4 | 11.5 | 11.37 | 25.46 | 0.136 | 10 | 7 |
| C | DJJO003 | *Dryophytes japonicus* | Seoguipo, Jeju | 33.23799 | 126.5478 | Brackish pool | 0.137 | 0.3 | 28.2 | 10.7 | 22.76 | 20.38 | 0.183 | 10 | 7 |
| C | DJJO004 | *Dryophytes japonicus* | Seoguipo, Jeju | 33.23796 | 126.5476 | Brackish pool | 0.798 | 0.4 | 30.4 | 13.7 | 28.38 | 20.69 | 0.198 | 15 | 7 |
| C | DJJO005 | *Dryophytes japonicus* | Seoguipo, Jeju | 33.23814 | 126.5474 | Brackish pool | 0.372 | 4 | 30.2 | 12.7 | 18.14 | 1.41 | 0.213 | 0 | 8 |
| C | DJJO006 | *Dryophytes japonicus* | Seoguipo, Jeju | 33.23866 | 126.547 | Brackish pool | 0.573 | 3.9 | 28.8 | 11 | 15.82 | 0.76 | 0.173 | 30 | 10 |
| C | DJJO007 | *Dryophytes japonicus* | Seoguipo, Jeju | 33.23858 | 126.547 | Brackish pool | 0.469 | 1.3 | 28.2 | 12.3 | 14.99 | 3.52 | 0.291 | 0 | 10 |
| C | DJJO008 | *Dryophytes japonicus* | Seoguipo, Jeju | 33.23867 | 126.5476 | Brackish pool | 0.138 | 2.2 | 28.4 | 11.7 | 8.99 | 5.22 | 0.198 | 0 | 11 |
| C | DJJO009 | *Dryophytes japonicus* | Seoguipo, Jeju | 33.23862 | 126.5476 | Brackish pool | 0.373 | 5.2 | 30.2 | 10.8 | 8.62 | 5.59 | 0.223 | 0 | 10 |
| D | DJJB001 | *Dryophytes japonicus* | Seoguipo, Jeju | 33.23602 | 126.3592 | Brackish pool | 3.745 | 0.3 | 29.5 | 12.7 | 3.81 | 49.93 | 0.274 | 10 | 4 |
| D | DJJB002 | *Dryophytes japonicus* | Seoguipo, Jeju | 33.23599 | 126.3591 | Brackish pool | 2.24 | 0.4 | 30.3 | 11.4 | 9.89 | 30.44 | 0.179 | 5 | 4 |
| D | DJJB003 | *Dryophytes japonicus* | Seoguipo, Jeju | 33.23596 | 126.3591 | Brackish pool | 0.831 | 0.4 | 28.2 | 13.5 | 4.54 | 28.81 | 0.551 | 5 | 3 |
| D | DJJB004 | *Dryophytes japonicus* | Seoguipo, Jeju | 33.23592 | 126.359 | Brackish pool | 1.261 | 0.1 | 30.3 | 11.7 | 8.35 | 28.24 | 0.227 | 5 | 3 |
| D | DJJB005 | *Dryophytes japonicus* | Seoguipo, Jeju | 33.23612 | 126.3593 | Brackish pool | 0.689 | 0.2 | 29.9 | 12.4 | 6.88 | 10.49 | 0.144 | 5 | 4 |
| D | DJJB006 | *Dryophytes japonicus* | Seoguipo, Jeju | 33.23622 | 126.3594 | Brackish pool | 0.436 | 0.2 | 29.3 | 12.5 | 8.52 | 15.23 | 0.158 | 5 | 4 |
| D | DJJB007 | Dryophytes japonicus | Seoguipo, Jeju | 33.23614 | 126.362 | Brackish pool | 0.538 | 0.2 | 31.2 | 12.2 | 8.44 | 43.31 | 0.586 | 0 | 3 |
| E | DJJC001 | *Dryophytes japonicus* | Seoguipo, Jeju | 33.3393 | 126.8495 | Pool | 0.029 | 0 | 30.8 | 11.9 | 216 | 33.09 | 0.061 | 5 | 3 |
| E | DJJC002 | *Dryophytes japonicus* | Seoguipo, Jeju | 33.3397 | 126.8492 | Pool | 0.032 | 0 | 29.7 | 11.5 | 279 | 19.5 | 0.167 | 10 | 4 |
| E | DJJC003 | *Dryophytes japonicus* | Seoguipo, Jeju | 33.3395 | 126.8493 | Pool | 0.02 | 0 | 29.9 | 11.2 | 256 | 22.65 | 0.153 | 10 | 4 |
| E | DJJC004 | *Dryophytes japonicus* | Seoguipo, Jeju | 33.3395 | 126.8492 | Pool | 0.053 | 0 | 29.8 | 11.2 | 258 | 30.5 | 0.091 | 10 | 4 |

**Table S3. Shapiro–Wilk normality test results for environmental variables before and after log-transformation.** Shapiro–Wilk W statistics present p-values for raw and log-transformed data, along with corresponding normality classifications. Temperature was the only variable that met normality assumptions in both raw and log-transformed form. Distance from the coastline and depth achieved normality only after log-transformation, while all other variables remained non-normal.

| **Variable** | **Shapiro_W_raw** | **Shapiro_p_raw** | **Shapiro_W_log** | **Shapiro_p_log** | **Normality_raw** | **Normality_log** |
| --- | --- | --- | --- | --- | --- | --- |
| Sal | 0.363 | < 0.001 | 0.9001 | < 0.001 | Non-normal | Non-normal |
| Temp | 0.9807 | 0.2468 | 0.9815 | 0.2763 | Normal | Normal |
| DO | 0.9562 | 0.0066 | 0.9597 | 0.0107 | Non-normal | Non-normal |
| DtC | 0.6881 | < 0.001 | 0.9809 | 0.2547 | Non-normal | Normal |
| DtF | 0.9102 | < 0.001 | 0.8997 | < 0.001 | Non-normal | Non-normal |
| Depth | 0.8973 | < 0.001 | 0.9883 | 0.6612 | Non-normal | Normal |
| Area | 0.1629 | < 0.001 | 0.8793 | < 0.001 | Non-normal | Non-normal |
| Elev | 0.9494 | 0.0026 | 0.8944 | < 0.001 | Non-normal | Non-normal |

**Table S4. Frequency of publications across ecological themes and habitat types for *Dryophytes japonicus*.** The frequency was measured by number of publications assigned to each ecological theme (behavior, breeding, conservation, ecology, hibernation, morphology, pathology, predation, and water quality/salinity) across four habitat categories documented in the literature dataset, agricultural land, artificial ponds, controlled conditions, and natural pools. These frequencies are used in the Chi-square test evaluating whether research themes are disproportionately associated with specific habitat types (see publication trends in Fig. 2).

| **Theme** | **Habitat type** | **Frequency** |
| --- | --- | --- |
| Behavior | Agricultural land | 6 |
| Breeding | Agricultural land | 8 |
| Conservation | Agricultural land | 5 |
| Ecology | Agricultural land | 25 |
| Hibernation | Agricultural land | 4 |
| Morphology | Agricultural land | 9 |
| Pathology | Agricultural land | 4 |
| Predation | Agricultural land | 6 |
| Water quality and salinity | Agricultural land | 2 |
| Behavior | Artificial pond | 0 |
| Breeding | Artificial pond | 0 |
| Conservation | Artificial pond | 0 |
| Ecology | Artificial pond | 3 |
| Hibernation | Artificial pond | 0 |
| Morphology | Artificial pond | 0 |
| Pathology | Artificial pond | 0 |
| Predation | Artificial pond | 0 |
| Water quality and salinity | Artificial pond | 0 |
| Behavior | Controlled condition | 3 |
| Breeding | Controlled condition | 2 |
| Conservation | Controlled condition | 1 |
| Ecology | Controlled condition | 2 |
| Hibernation | Controlled condition | 2 |
| Morphology | Controlled condition | 0 |
| Pathology | Controlled condition | 0 |
| Predation | Controlled condition | 2 |
| Water quality and salinity | Controlled condition | 0 |
| Behavior | Natural pool | 1 |
| Breeding | Natural pool | 1 |
| Conservation | Natural pool | 0 |
| Ecology | Natural pool | 5 |
| Hibernation | Natural pool | 0 |
| Morphology | Natural pool | 0 |
| Pathology | Natural pool | 0 |
| Predation | Natural pool | 3 |
| Water quality and salinity | Natural pool | 1 |

**Table S5. Frequency of publications across ecological themes and decade categories.** The frequency was measured by number of studies published within each ecological theme across four historical decade categories (1931–1950, 1971–1990, 1991–2010, and 2011–2023). These frequencies were used in the Chi-square test evaluating whether thematic research focus has shifted over time.

| **Theme** | **Decade category** | **Frequency** |
| --- | --- | --- |
| Behavior | 1931-1950 | 0 |
| Breeding | 1931-1950 | 1 |
| Conservation | 1931-1950 | 0 |
| Ecology | 1931-1950 | 0 |
| Hibernation | 1931-1950 | 0 |
| Morphology | 1931-1950 | 0 |
| Pathology | 1931-1950 | 0 |
| Predation | 1931-1950 | 0 |
| Water quality and salinity | 1931-1950 | 0 |
| Behavior | 1971-1990 | 0 |
| Breeding | 1971-1990 | 0 |
| Conservation | 1971-1990 | 1 |
| Ecology | 1971-1990 | 0 |
| Hibernation | 1971-1990 | 0 |
| Morphology | 1971-1990 | 1 |
| Pathology | 1971-1990 | 0 |
| Predation | 1971-1990 | 1 |
| Water quality and salinity | 1971-1990 | 0 |
| Behavior | 1991-2010 | 4 |
| Breeding | 1991-2010 | 2 |
| Conservation | 1991-2010 | 1 |
| Ecology | 1991-2010 | 6 |
| Hibernation | 1991-2010 | 1 |
| Morphology | 1991-2010 | 1 |
| Pathology | 1991-2010 | 0 |
| Predation | 1991-2010 | 2 |
| Water quality and salinity | 1991-2010 | 0 |
| Behavior | 2011-2023 | 6 |
| Breeding | 2011-2023 | 8 |
| Conservation | 2011-2023 | 4 |
| Ecology | 2011-2023 | 29 |
| Hibernation | 2011-2023 | 5 |
| Morphology | 2011-2023 | 7 |
| Pathology | 2011-2023 | 4 |
| Predation | 2011-2023 | 8 |
| Water quality and salinity | 2011-2023 | 3 |

**Table S6. Frequency of publications across habitat types and decade.** The number of studies reporting *Dryophytes japonicus* across four habitat types, agricultural land, artificial ponds, controlled conditions, and natural pools, within each decade category represented in the literature dataset (1931–1950, 1971–1990, 1991–2010, and 2011–2023). These frequencies are used in the Chi-square test evaluating whether habitat use in published studies shows any temporal pattern or disproportionate representation across historical periods.

| **Habitat type** | **Decade category** | **Frequency** |
| --- | --- | --- |
| Agricultural land | 1931-1950 | 1 |
| Artificial pond | 1931-1950 | 0 |
| Controlled condition | 1931-1950 | 0 |
| Natural pool | 1931-1950 | 0 |
| Agricultural land | 1971-1990 | 1 |
| Artificial pond | 1971-1990 | 0 |
| Controlled condition | 1971-1990 | 1 |
| Natural pool | 1971-1990 | 1 |
| Agricultural land | 1991-2010 | 13 |
| Artificial pond | 1991-2010 | 1 |
| Controlled condition | 1991-2010 | 3 |
| Natural pool | 1991-2010 | 0 |
| Agricultural land | 2011-2023 | 54 |
| Artificial pond | 2011-2023 | 2 |
| Controlled condition | 2011-2023 | 8 |
| Natural pool | 2011-2023 | 10 |

**Table S7. Comparison of Akaike information criterion (AIC) among candidate GLMs used to model breeding microhabitat selection in *Dryophytes japonicus*.** The AIC values are shown for the null GLM, the full GLM including all predictors, and the final model selected using stepwise backward selection (step() function).

| **Model** | **AIC** |
| --- | --- |
| Null GLM | 106.12 |
| Full GLM | 94.64 |
| Stepwise-selected GLM | 89.52 |

**Table S8. Summarized eigenvalues and variance explained by principal components.** The eigenvalues, proportion of variance explained, and cumulative variance for each principal component (PC1–PC8) derived from the PCA of environmental and topographic variables measured across the coastal breeding pools. PC1 and PC2 together explain 40.01% of the total variance, with subsequent components contributing progressively smaller proportions.

| **PC** | **Eigenvalue** | **Proportion** | **Cumulative** |
| --- | --- | --- | --- |
| PC1 | 1.7845 | 0.2231 | 0.2231 |
| PC2 | 1.4167 | 0.1771 | 0.4001 |
| PC3 | 1.329 | 0.1661 | 0.5663 |
| PC4 | 0.9609 | 0.1201 | 0.6864 |
| PC5 | 0.7693 | 0.0962 | 0.7825 |
| PC6 | 0.6837 | 0.0855 | 0.868 |
| PC7 | 0.6551 | 0.0819 | 0.9499 |
| PC8 | 0.4008 | 0.0501 | 1 |

**Table S9.** **The loading values for each environmental and topographic variable on PC1–PC3 from the principal component analysis.** Positive or negative loadings indicate the direction and strength of each variable’s contribution to the corresponding principal component. Variables with larger absolute loadings exert stronger influence on that component’s multivariate structure. The variables included are salinity, temperature, dissolved oxygen, distance to coastline, distance to forest, pool depth, pool area, and elevation.

| **Variable** | **PC1** | **PC2** | **PC3** |
| --- | --- | --- | --- |
| Salinity | 0.4202 | -0.1301 | 0.3097 |
| Temperature | 0.0757 | -0.1791 | -0.6349 |
| Dissolved Oxygen | -0.2075 | -0.395 | 0.4964 |
| Distance to coastal | -0.4224 | 0.2847 | -0.319 |
| Distance to forest | 0.6496 | -0.0359 | -0.0664 |
| Depth of pool | -0.093 | -0.6336 | -0.1497 |
| Area of pool | -0.0774 | -0.5533 | -0.2429 |
| Elevation of pool | -0.3995 | -0.0704 | 0.2588 |

**Table S10. The performance statistics of the Random Forest classification model used to predict tadpole occurrence.** Metrics include the number of trees grown (ntree), the number of variables randomly sampled at each split (mtry), the out-of-bag (OOB) error rate, and the overall model accuracy. An OOB error rate of 0.2451 corresponds to an overall accuracy of 0.7549, indicating that the model correctly classified approximately 75% of unseen data.

| **Metric** | **Value** |
| --- | --- |
| ntree | 500 |
| mtry | 3 |
| OOB_error_rate | 0.2451 |
| Overall_accuracy | 0.7549 |

**Table S11. MeanDecreaseGini values for each environmental and topographic predictor used in the Random Forest classification model (Table S6; Fig. 5).** Higher values indicate greater contribution to model performance. Distance of pool from coastline and distance of pool from forest were the strongest predictors of tadpole occurrence, followed by pool depth, pool area, salinity, elevation, dissolved oxygen, and temperature. Site identity had the lowest importance among predictors.

| **Variable** | **MeanDecreaseGini** |
| --- | --- |
| DtC | 5.835 |
| DtF | 5.438 |
| Depth | 4.941 |
| Area | 4.762 |
| Sal | 4.04 |
| Elev | 3.393 |
| DO | 3.348 |
| Temp | 3.335 |
| Site | 1.508 |

**Supplementary Figures**


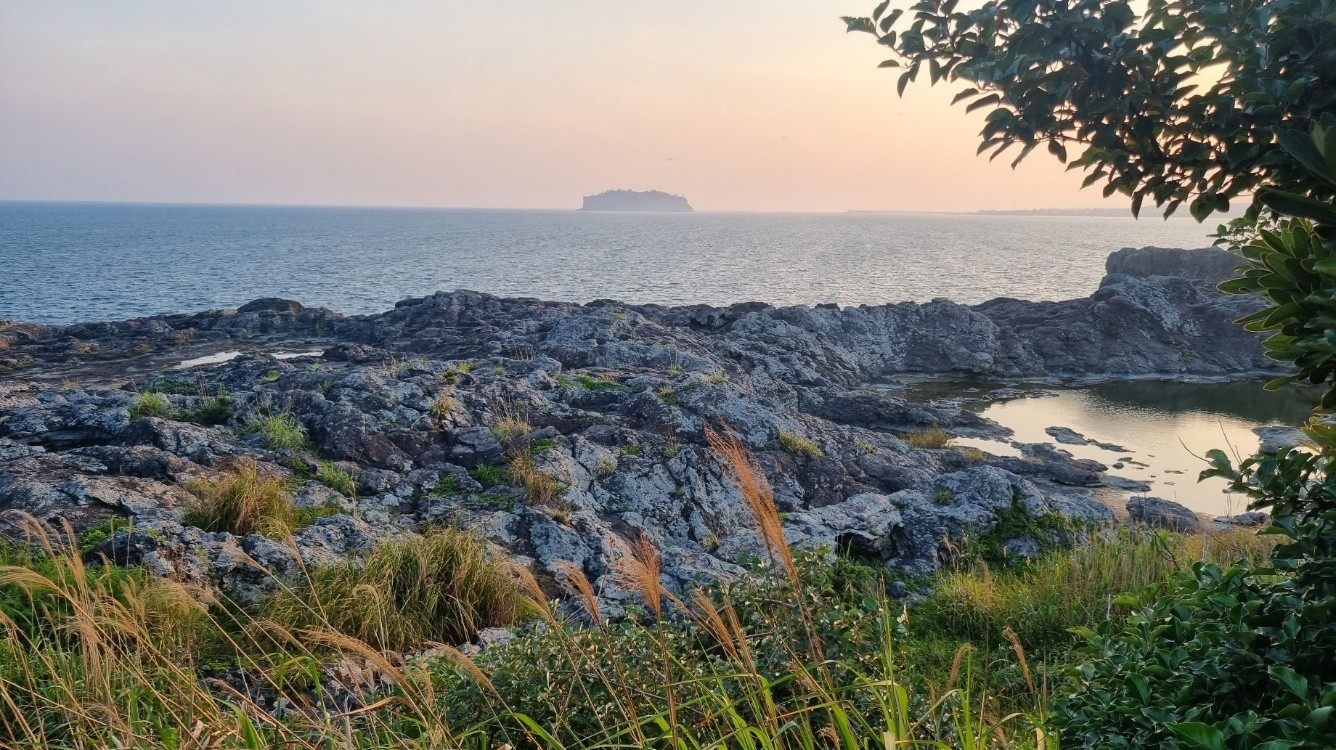


**Fig. S1. Coastal breeding habitat of *Dryophytes japonicus* at Sae Island (Site B).** The majority of coastal pools containing tadpole occurrences were found at this site.

**
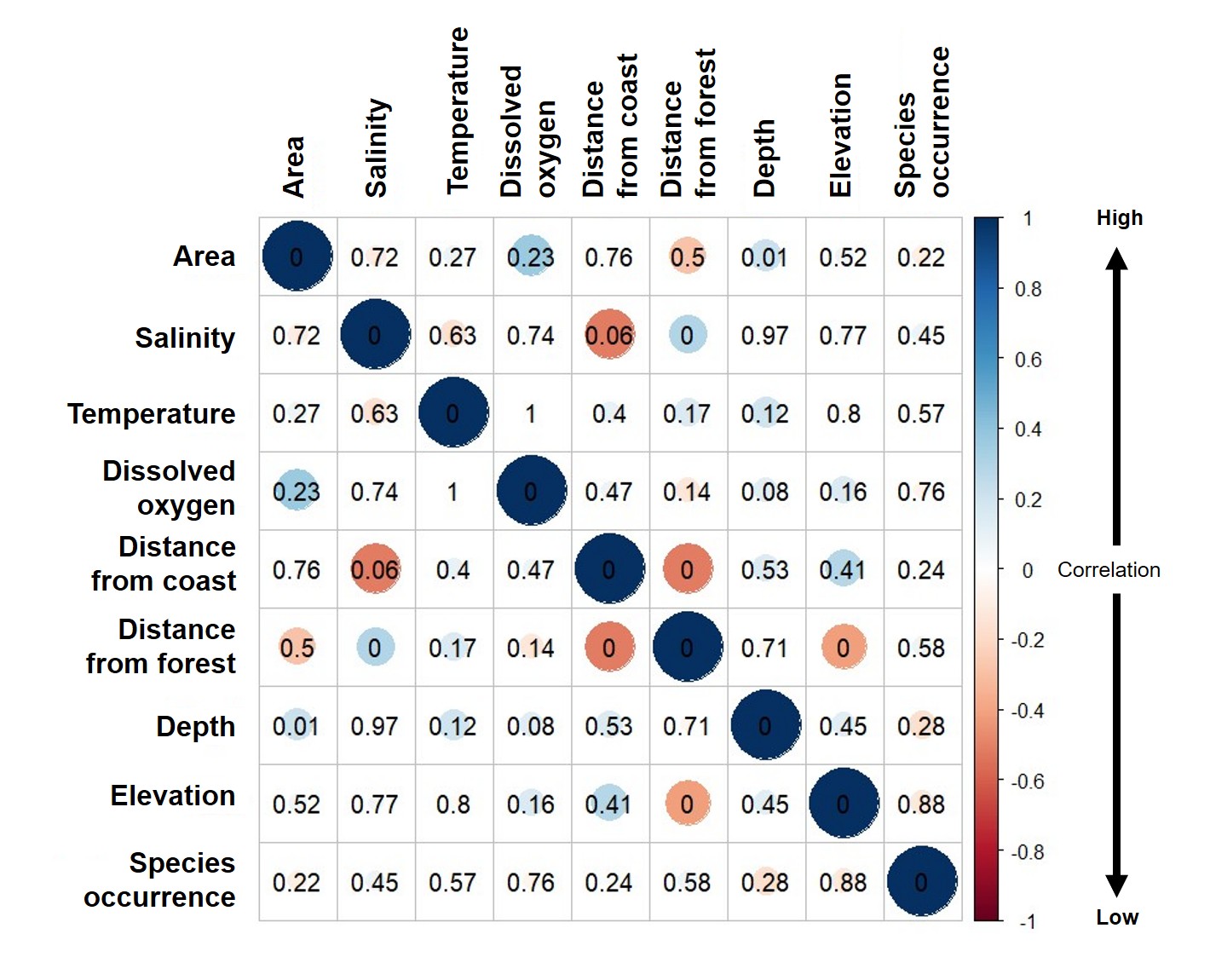
**

**Fig. S2. Correlation matrix of environmental variables and species occurrence.** Salinity, dissolved oxygen, temperature, and elevation were highly positively correlated (r = 0.63–0.97). Pool area was positively associated with distance from coastline and elevation, while depth strongly correlated with salinity (r = 0.97). Species occurrence showed positive correlations with dissolved oxygen (r = 0.76) and elevation (r = 0.88). Negative or weak relationships were found for distance from forest and coastline. These results indicate strong environmental covariation among coastal, physicochemical, and structural variables.
